# High-dimensional multi-pass flow cytometry via spectrally encoded cellular barcoding

**DOI:** 10.1038/s41551-023-01144-9

**Published:** 2023-11-30

**Authors:** Sheldon J. J. Kwok, Sarah Forward, Marissa D. Fahlberg, Emane Rose Assita, Sean Cosgriff, Seung Hyung Lee, Geoffrey R. Abbott, Han Zhu, Nicolas H. Minasian, A. Sean Vote, Nicola Martino, Seok-Hyun Yun

**Affiliations:** 1 LASE Innovation Inc., Woburn, MA, USA.; 2 Harvard Medical School and Wellman Center for Photomedicine, Massachusetts General Hospital, Cambridge, MA, USA.

## Abstract

Advances in immunology, immuno-oncology, drug discovery and vaccine development demand improvements in the capabilities of flow cytometry to allow it to measure more protein markers per cell at multiple timepoints. However, the size of panels of fluorophore markers is limited by overlaps in fluorescence-emission spectra, and flow cytometers typically perform cell measurements at one timepoint. Here we describe multi-pass high-dimensional flow cytometry, a method leveraging cellular barcoding via microparticles emitting near-infrared laser light to track and repeatedly measure each cell using more markers and fewer colours. By using live human peripheral blood mononuclear cells, we show that the method enables the time-resolved characterization of the same cells before and after stimulation, their analysis via a 10-marker panel with minimal compensation for spectral spillover and their deep immunophenotyping via a 32-marker panel, where the same cells are analysed in 3 back-to-back cycles with 10–13 markers per cycle, reducing overall spillover and simplifying marker-panel design. Cellular barcoding in flow cytometry extends the utility of the technique for high-dimensional multi-pass single-cell analyses.

Fluorescence-based flow cytometry has been a workhorse in the single-cell analysis of surface markers, intracellular cytokines, intranuclear proteins (such as transcription factors) and cell cycle. Continuing advances in high-speed fluidics and multicolour optics, as well as fluorophore chemistry, has enabled high-parameter measurement (up to ~40 markers) at high speed (>10,000 cells per second) and low cost^1,2^. While these are major advantages in throughput and cost over technologies such as single-cell mass cytometry^3^ and sequencing-based proteomic analysis^4^, flow cytometry is facing substantial challenges in meeting the growing demand to measure more protein markers per cell. Highly multiplexed measurements of immune cells to characterize dozens of different cell types have proven to be critical in the development of immunotherapies and vaccines^5–7^, as well as in the detection of minimal residual disease in leukaemia^8^. However, high-marker analysis (>30 protein markers) is challenging due to the ambiguity caused by spectral spillover between fluorophores, often requiring months-long optimization of fluorophore–antibody combinations and instrument settings^9,10^. Clinical laboratories that have the labour and time available to optimize a high-marker panel may still lack the expertise to design and select the appropriate reagents. Limited availability of well-validated fluorophores (colours) is an additional barrier, especially for clinical applications that require the use of reagents that meet US Food and Drug Administration (FDA) manufacturing requirements. For these reasons, most clinical laboratories use standardized panels for immunophenotyping with up to ~10 colours, which restricts the types of cell that can be detected at once and increases the number of cell samples required^11–15^.

A major advantage of flow cytometry is that, unlike mass cytometry and sequencing, cells are not destroyed during optical acquisition. Flow sorters rely on this non-destructive feature. However, current flow cytometer ‘analysers’ are typically used for one-time measurement of cells. Measuring cells twice using a flow sorter is in principle possible, but single-cell information would be lost in the cell collection process. This limitation, which has not been openly recognized, makes current flow cytometry unable to address the ever growing need to acquire high-dimensional data and temporal responses of single cells^16,17^.

Here we introduce a new approach in flow cytometry that leverages the optical barcoding of individual cells. Barcoding techniques have been used previously in cytometry for tracking different samples, enabling the pooling of samples for faster analysis. These techniques, relying on fluorescence intensity differences in flow cytometry^18^ or on a limited set of radioisotopes in mass cytometry^19^, are only suitable for tracking tens of samples at a time. Here we use laser particles (LPs), recently developed laser light-emitting microparticles^20^, to tag and track up to millions of cells at a time. This approach enables multi-pass flow cytometry in which the same cells are measured multiple times using each cell’s unique optical barcodes to align and concatenate data from different measurements. We used this method to acquire flow cytometry data from human peripheral blood mononuclear cells (PBMCs). First, we applied our multi-pass approach to a common assay involving in vitro stimulation of T cells in PBMCs. We demonstrate unprecedented characterization of the same T cells before and after stimulation, enabling quantification of biomarker downregulation. Second, we report a 10-marker panel on live T cells requiring minimal compensation. Finally, we applied multi-pass cytometry to high-marker analysis. We developed a broad immunophenotyping panel optimized for 3 measurement cycles of leucocyte populations. Our ‘cyclic’ approach greatly simplifies high-parameter analysis by requiring a far fewer number of fluorophores for the same number of markers. We performed this 32-marker assay on live human PBMCs from a healthy donor and validated our results against published data.

## Results

### Multi-pass flow cytometer instrumentation

**Fig. 1 |:**
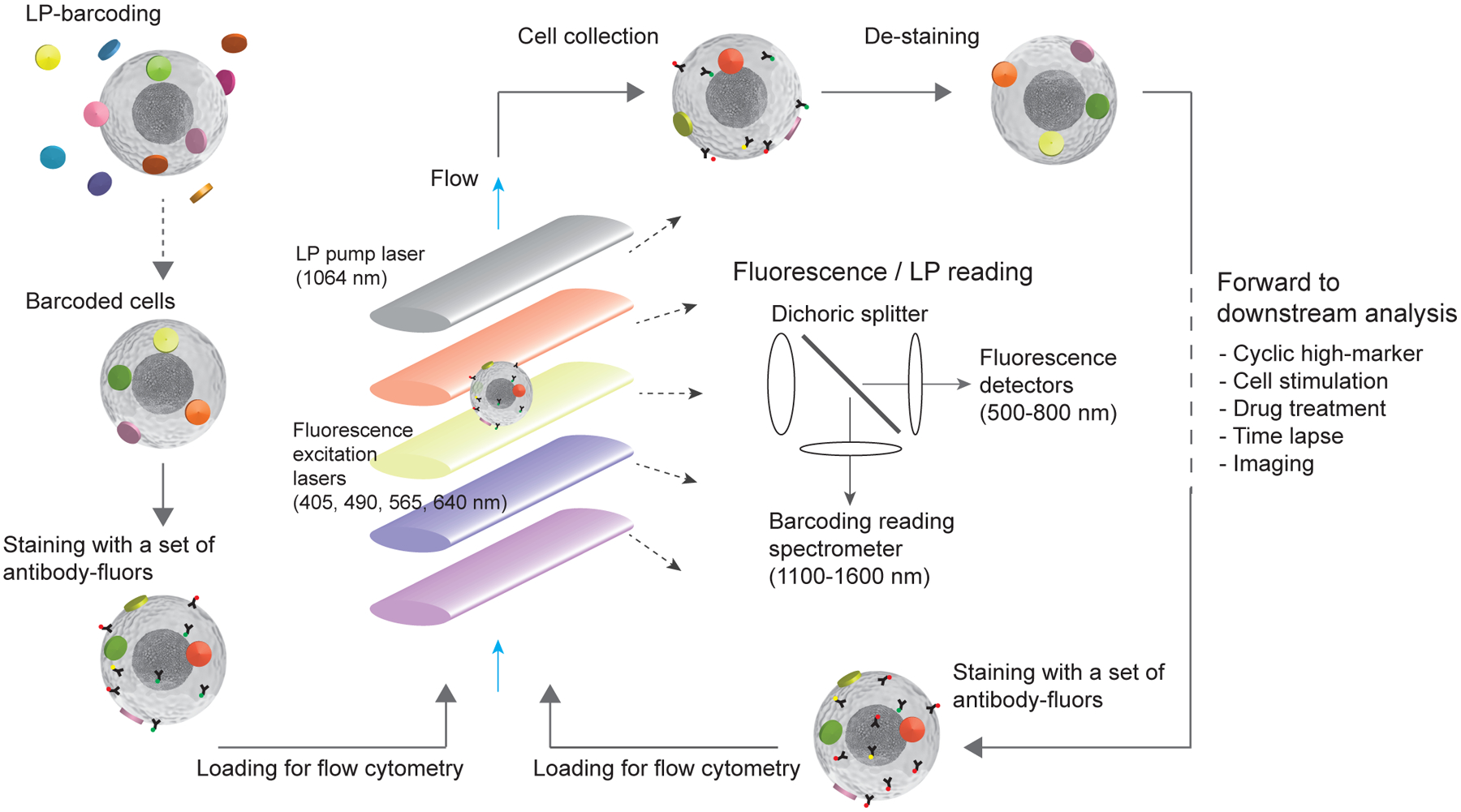
Schematic of multi-pass flow cytometry. The major steps include: tagging cells with LPs to yield LP-barcoded cells which typically have 3+ LPs; staining cells with a first set of fluorophore-conjugated antibodies; loading the barcoded and stained cells into a flow cytometer that detects fluorescence signals using multiple excitation lasers and LP lasing signals using a pump laser and spectrometer; then collecting the cells, destaining (for example, photobleaching of the fluorophores or chemical release of antibodies), restaining the cells with another set of antibodies and measuring them again in the flow cytometer. In addition to flow cytometry, this technology can be expanded to further downstream and upstream analysis of the barcoded cells.

Figure 1 illustrates the general workflow of multi-pass flow cytometry along with the optical measurements of cellular barcodes and fluorescent reagents. First, cells are mixed with excess LPs in solution to label each cell with a unique, random combination of LPs. Next, cells are stained with a first set of antibody-fluorophores and then loaded into a flow cytometer capable of exciting and detecting the laser emission from LPs and also collecting the cells after the flow measurement. We built such a multi-pass flow cytometer using a near-infrared (NIR) pump laser (1,064 nm) to stimulate the laser emission of LPs and four fluorescence excitation lasers (405 nm, 488 nm, 561 nm and 638 nm) to elicit fluorescence. Cells flow across the laser beams in a hydrodynamically focused stream at a velocity of ~3.4 m s^−1^. The fluorescence signal is split by dichroic filters and detected by avalanche photodiodes, while the lasing signal is detected by a line-scan spectrometer using a 2,048-pixel InGaAs charge-coupled device (CCD). Following data acquisition, the antibody-fluorophores in the collected cells are deactivated by either the photobleaching of the fluorophores or the release of the antibodies from the cells. For the next cycle of measurement, the cells are stained with a subsequent set of antibody-fluorophores and loaded back into the flow cytometer.

**Fig. 2 |:**
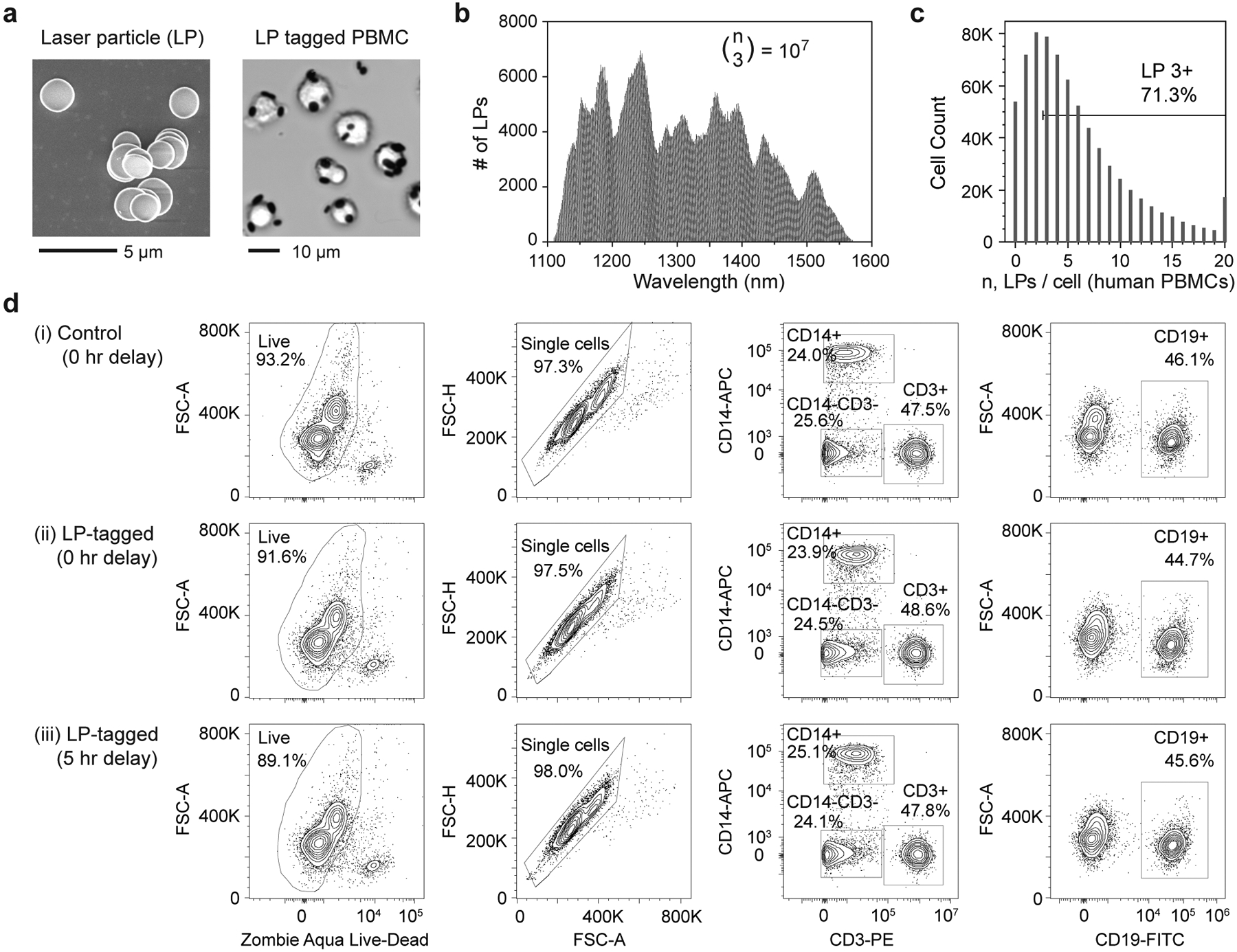
Tagging of human PBMCs with LPs. **a**, Left: scanning electron micrograph of polymer-silica-coated LPs. Right: optical image of live human PBMCs tagged with LPs. **b**, Lasing wavelength distribution of the LPs comprising 10^7^ distinguishable barcodes when used in combinations of 3 or more. **c**, Histogram showing distribution of LPs per cell, with over 70% of cells having 3 or more LPs. **d**, Immunophenotyping data comparing cell viability (left column), singlet purity (middle left column) and frequency of monocytes, T cells and B cells (middle right and right columns) for (**i**) control, untagged cells at 0 h, (**ii**) LP-tagged cells at 0 h and (**iii**) LP-tagged cells at 5 h post tagging.

As described previously, we employed LPs made of InGaAsP microdiscs^20^ that are 1.6–1.9 μm in diameter and 220–290 nm in thickness (Fig. 2a). Six different compositions of bulk In_x_Ga_1-x_As_y_P_1-y_ epitaxial layers were used to ensure that each LP emits a lasing peak between 1,150 and 1,550 nm (Fig. 2b), which leaves the entire visible and NIR-I (700–900 nm) wavelength ranges free for fluorescence labelling. We first coated the semiconductor microdiscs with a ~50 nm layer of SiO_2_ to ensure stability and confer biocompatibility. We have previously shown that silica-coated LPs can be internalized into a variety of cell types with overnight incubation^20^. To shorten the tagging time, we functionalized the silica coating surface with polyethylenimine (PEI), a cationic polymer known to bind to cell membranes^21^. We found that PEI-silica-coated LPs were efficiently attached to live human PBMCs within 15 min of mixing and centrifugation (Fig. 2a). A given cell is defined as barcoded if it is tagged with 3 or more LPs. By mixing an excess of LPs to cells, typically 70% of PBMCs can be tagged with 3 or more LPs (Fig. 2c). We also developed an antibody-based method for tagging LPs to cells through biotin–streptavidin coupling, which enables barcoding of specific cell types. This approach requires initial staining of cells with biotinylated antibodies for suitable surface antigens, similar to cell hashing methods^22^. Both tagging approaches target LP binding to the cell surface. The LPs remain attached on the cell surface of most lymphoid cells, while myeloid cells and epithelial cells tend to internalize LPs through macropinocytosis^20,23^. With either cationic coating or antibody tagging, an event detected on our cytometer with 3 or more LPs can be classified as originating from a cell, which can be used to distinguish cells from debris or free LPs (similar to CD45 for immune cells).

Using a viability dye that assesses membrane integrity, we found that cell viability is nearly unchanged from 93.2% before to 91.6% after LP tagging. Storing the LP-tagged samples in standard wash buffer at 4 °C for 5 h reduces viability slightly further to 89.1% (Fig. 2d). There was no measurable difference in singlet purity between control and LP-tagged samples at 0 and 5 h, indicating that LP tagging does not increase cell–cell aggregation. To test whether LP tagging affects cellular phenotypes, we also compared the expression of major immune markers including CD45 (pan immune marker), CD14 (monocyte marker), CD3 (T-cell marker) and CD20 (B-cell marker). There were no significant differences in the population percentages of any of these markers when comparing control and LP-tagged samples at 0 and 5 h (Fig. 2d). Furthermore, using CD3-KromeOrange, we found that the median fluorescence intensity decreased ~5% for cells tagged with 3–5 LPs and ~15% for cells tagged with 10 LPs (Supplementary Fig. 1).

### Repeated measurement of the same cells

**Fig. 3 |:**
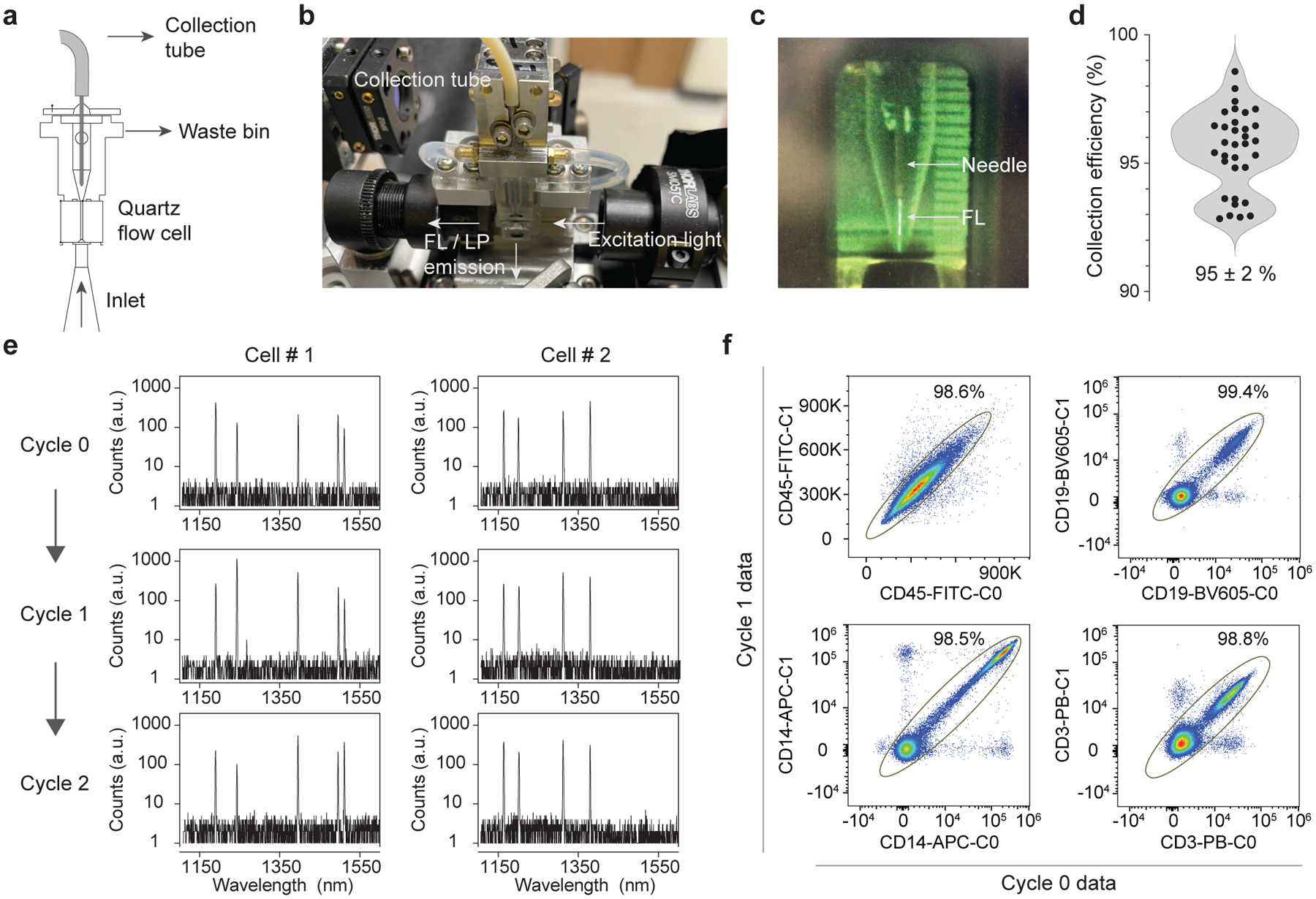
Repeated measurements of the same cells. **a**,**b**, Schematic (**a**) and image (**b**) of modified fluidics to incorporate a needle and collection tube for cell capture. **c**, Fluorescein dye (FL) flowing into the collection needle. **d**, Cell collection efficiency of live CD45^+^ human PBMCs over 32 trials of capturing cells using the modified flow cell, with mean and s.d. of 95% ± 2%. **e**, Representative lasing spectra of two LP-tagged cells measured repeatedly over 3 cycles. An algorithm was used to match cells between measurements using the lasing wavelengths. **f**, Validation of matching LP spectral barcodes. LP-tagged cells stained with antibody-fluorophores were measured in successive cycles (Cycle 0 and Cycle 1) and then matched using their lasing spectra. Plots show strong correlation between fluorescence signals measured in the two cycles.

Cells are collected after each measurement so that the same cells can be measured again in the subsequent cycle. In conventional flow cytometers employing hydrodynamic focusing, cells flow through a glass flow cell along with sheath fluid and are then diverted into waste following analysis. We developed a cell-collecting fluidic channel that recovers all the cells in the focused core stream of 10–20 μm width at the exit of the flow cell (Fig. 3a,b). The collection channel consists of a 127-μm diameter needle, followed by a polypropylene-based flexible tube connected to a peristaltic pump that controls the flow rate of the collected stream (Fig. 3c). With sample input flow rates of 30 μl min^−1^, a sheath flow rate of 9 ml min^−1^ and a collection flow rate of ~400 μl min^−1^, we were able to collect nearly 100% of the input cells into a tube. To ensure viability of cells in the tube during flow acquisition, we used phosphate-buffered saline (PBS) as the sheath fluid, and the cells were collected in serum-supplemented buffer. Immediately after acquisition, the cells are washed and resuspended in standard flow cytometry staining buffer or a viability dye solution. Overall, the cell collection and washing process typically recovers 95% ± 2% of live human PBMCs (Fig. 3d). There was no change in the viability of human PBMCs after 1, 2 or 3 cycles of cell capture (Supplementary Fig. 2).

Cells measured in different passes were matched using their LP barcodes. Our spectrometer measures the lasing spectra with resolution of ~0.5 nm. Given that single LPs provide ~800 distinguishable lasing peaks (from 1,150 to 1,550 nm), a combination of 3 random LPs per cell in principle can provide _800_C_3_ = 8.5 × 10^7^ unique spectral barcodes, sufficient for tracking a population of 1,000,000 cells with <2% of duplication-induced error (loss). LP barcodes measured in different passes were matched by extracting the peak wavelengths and computing the probability of a match by comparing pairs of measured spectra. Each potential match is scored depending on the lasing wavelengths detected and their emission amplitudes (see Methods). Examples of matched spectra over three cycles are given in Fig. 3e. We validated our approach by staining LP-tagged PBMCs with major immune markers (CD45, CD14, CD3 and CD19), measuring the same cells twice using the modified flow cytometer and comparing the fluorescence data of cells that were matched using the LP barcodes. We defined an apparently correctly matched population in which the fluorescence intensities of the same cells measured in Cycle 0 and Cycle 1 are strongly correlated (Fig. 3f). This population ranged from 98.5% to 99.4% of the cells. The remaining 0.6–1.5% of cells that were apparently correctly matched appear as noise and do not meaningfully affect data quality and resolution. We further validated our approach by verifying whether LP barcodes could be used to keep track of sample identity. Data from three separately acquired samples were pooled together and matched. Less than 2% of the matched cells were erroneously identified as belonging to two different samples (Supplementary Fig. 3). Our validation data show that our approach can track and match cells between different measurements with high accuracy.

### Time-resolved measurements of T-cell activation

One potentially impactful application of multi-pass flow cytometry is time-lapse flow analysis. Multi-pass flow cytometry can be used to measure changes in marker expression of individual cells between subsequent cycles due to various biological processes either naturally occurring or artificially induced, such as cell division, incubation, drug treatments, cell–cell interactions or stimulations. To measure the same markers in successive measurements, we employed chemically releasable antibodies^24^ (REAlease, Miltenyi Biotec), which are designed to be released from live cells by the addition of a chemical reagent.

We explored time-resolved flow cytometry analysis of T cells upon cell stimulation. Drug treatments or immunotherapies can alter the expression of protein markers on certain cell types, reflecting changes in activation state, viability, drug response or resistance^25,26^. While conventional flow cytometry can compare population differences between treated and untreated cells, it cannot identify changes to each individual cell, which is especially important for heterogeneous cell samples. In addition, changes in marker expression can prevent or impair identification of cell type post stimulation^27^. With a one-time measurement, it is difficult to distinguish between processes such as downregulation, upregulation, proliferation or cell death, especially when multiple cell types are present in the sample.

**Fig. 4 |:**
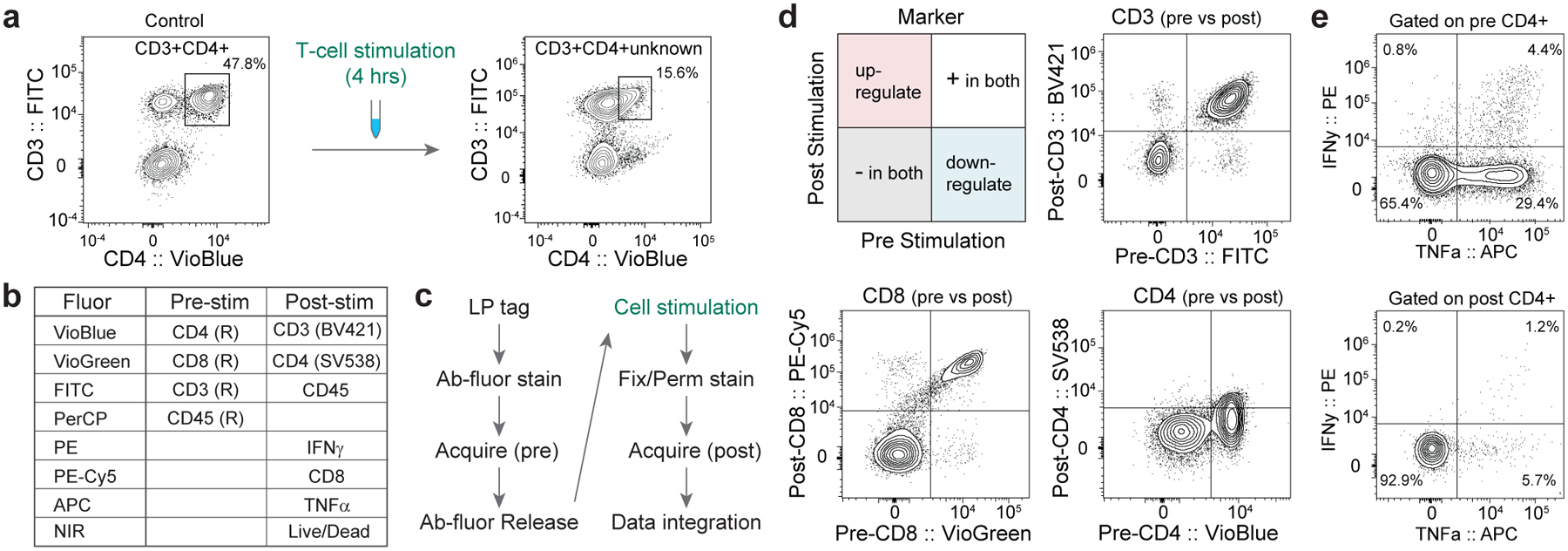
Time-resolved flow cytometry. **a**, Conventional flow cytometry can identify CD3^+^CD4^+^ cells pre-stimulation (left), but T-cell stimulation results in loss of CD4 signal, making it difficult to identify CD3^+^CD4^+^ cells post stimulation (right). **b**, Panel design of time-lapse flow cytometry assay. Releasable antibodies are denoted by (R). **c**, Schematic of workflow in which T cells are first stained with pre-stim antibodies and then acquired for phenotyping. Releasable antibodies are then removed and the cells are stimulated. Finally, the cells undergo fixation/permeabilization, staining with post-stim antibodies and are acquired again. **d**, Plotting marker signals measured pre and post stimulation enables identification of markers that are upregulated or downregulated (top left). Plotting CD3 pre vs CD3 post revealed minimal change with stimulation (top right). Plotting CD8 pre vs CD8 post also revealed minimal change with stimulation (bottom left). Plotting CD4 pre vs CD4 post revealed significant downregulation (bottom right). **e**, Gating on pre-stimulation CD4^+^ cells enables identification of cytokine-secreting cells (top). Gating on post-stimulation CD4^+^ cells identifies significantly fewer cytokine-secreting cells due to CD4 downregulation (bottom).

Using a basic T-cell panel, we measured two different samples of T cells in human PBMCs from the same donor with and without stimulation with phorbol myristate acetate (PMA) and ionomycin, which is widely used to determine the potential function of immune cells^28–30^. As shown in Fig. 4a, while CD4^+^CD3^+^ T cells can readily be identified before stimulation, CD4 expression is substantially reduced post stimulation, making it difficult to re-identify these cells. Furthermore, it is difficult with conventional flow cytometry to distinguish whether changes in markers are phenotypic switches or are due to expansion or death of specific cell types.

For time-resolved characterization of T-cell stimulation, human PBMCs were tagged with LPs via antibody binding, stained with chemically releasable antibodies (CD3, CD8 and CD4) and analysed for baseline phenotyping (before stimulation) (Fig. 4b,c). After the first acquisition, the releasable antibodies were removed and the cells were stimulated for 4 h with PMA/ionomycin. The cells were then restained for the same surface markers as well as intracellular cytokines (after fixation/permeabilization), for a second post-stimulation acquisition. Figure 4d shows expression of different markers (CD3, CD8 and CD4) pre and post stimulation for each cell. This pre-and-post comparison plot enables quantitative analysis of the degree of downregulation (bottom-right quadrant) for each cell that is measured. While CD8 and CD3 expression were relatively unchanged, we identified loss of CD4 expression on T cells that was otherwise not easily identifiable with a single time-point measurement. Our time-resolved approach enables gating on pre-stimulation markers for downstream analysis, such as identification of cytokine-secreting cells that were CD4^+^ before stimulation (Fig. 4e). We also verified that our LP-barcoding approach does not appreciably affect secretion of IFNγ and TNFα from T cells (Supplementary Fig. 4).

### 10-marker, 2-cycle characterization of T cells

**Fig. 5 |:**
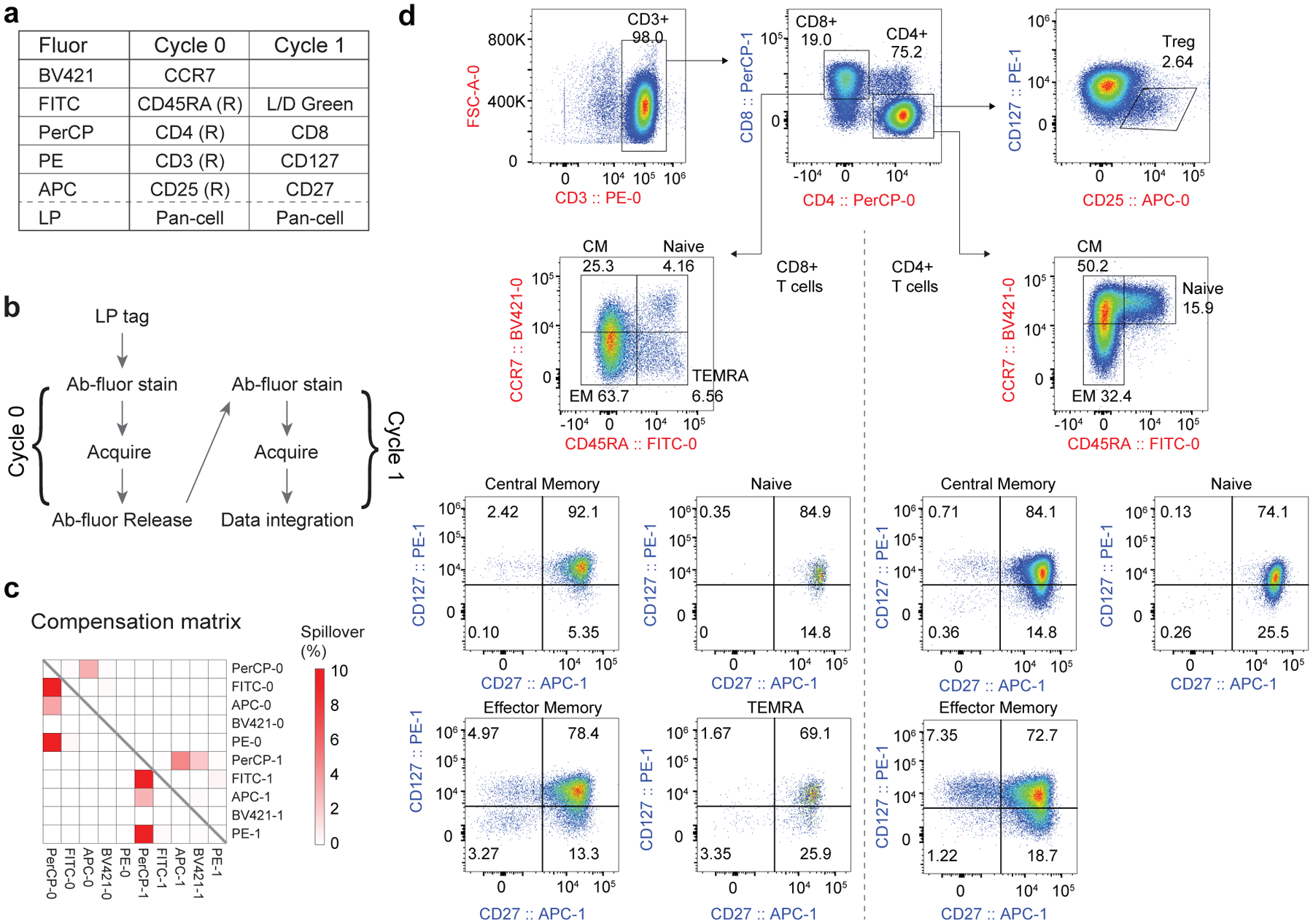
10-marker multi-pass panel using releasable antibodies. **a**, Two-cycle panel design using releasable antibodies (R) and conventional antibodies. **b**, Schematic of workflow for this panel. Cells were tagged and stained with Cycle 0 before flow cytometry acquisition and cell capture. Releasable antibodies were removed and the process was repeated with conventional antibodies shown in Cycle 1. **c**, Compensation matrix showing compensation values from both cycles concatenated into one matrix. **d**, Representative gating strategy showing identification of T_regs_ (top right), memory CD4 (right of dashed line) and CD8 (left of dashed line) subtypes, and corresponding expression of CD27 and CD127 on each subtype. CM, central memory; EM, effector memory.

LP barcoding allows us to acquire different markers over multiple cycles and integrate the data all together. Additional markers can be measured on the same cells without the need of additional fluorophores that complicate panel design through spectral spillover. We explored whether this multi-pass approach can enable 10-marker analysis of T cells with minimal spectral spillover. Isolated live human T cells were barcoded with PEI-LPs and then stained with a Cycle-0 panel of 5 releasable antibodies (Fig. 5a). After staining, the cells were acquired and captured, releasable antibodies were removed and cells were restained with a new Cycle-1 panel of 5 markers (Fig. 5b) before being acquired a second time. The resulting 10-marker compensation matrix includes only 9/90 (10%) fluorophore pairs with compensation >1%, 50 pairs with zero spillover at all (0.0% compensation) and 20 pairs with compensation <0.1% (Fig. 5c). This level of spillover is substantially lower than those of conventional (single-pass) 10-marker panels using 10 different fluorophores^31,32^. Memory T-cell populations including T_regs_, central memory, naïve, effector memory and TEMRA were all clearly identifiable, and cells were further subset by differential CD27 and CD127 expression (Fig. 5d). Repeating this assay several times yielded coefficients of variation (c.v.s) <30%^33–35^ (Supplementary Fig. 5).

### Photobleaching of common fluorophores

To measure different markers in each pass, there is a need to either remove antibodies or inactivate fluorescence signals after each measurement so that the cells can be restained with a different set of fluorophore-conjugated antibodies. While chemically releasable antibodies are suitable, the limited portfolio of antibodies commercially available makes it difficult to use for high-marker panels. There are also a number of approaches for antibody stripping and/or iterative staining on fixed cells^36–39^ (CODEX, CyCIF, IBEX and 4i), but fewer well-validated options exist for live cells.

**Fig. 6 |:**
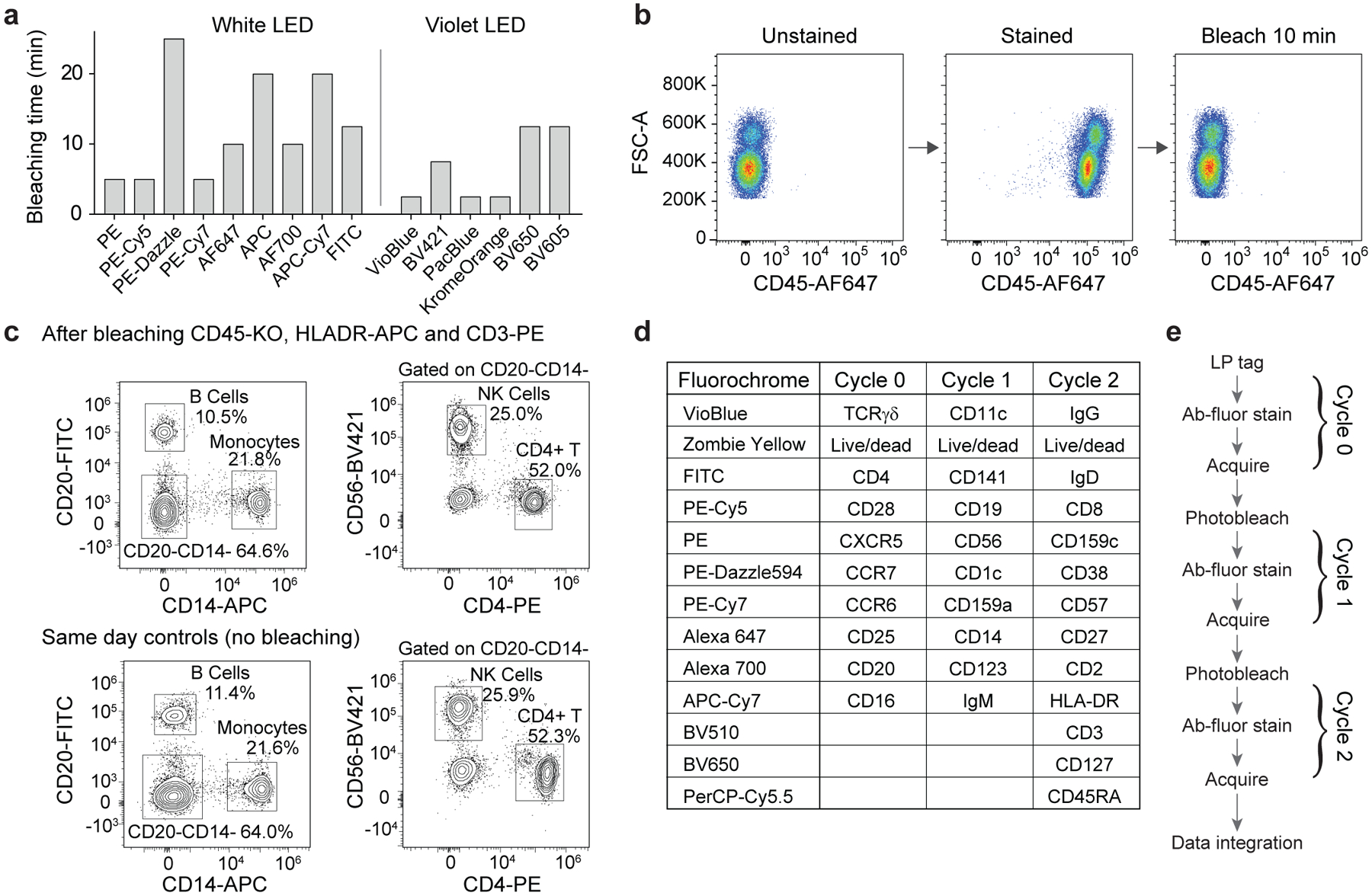
Fluorophore photobleaching for high-marker multi-pass cytometry. **a**, Photobleaching time for fluorophores conjugated to CD45 excited by a broad-spectrum white LED or a 405 nm LED. **b**, Representative data showing complete fluorescence signature erasure via photobleaching. Cells stained with CD45-AF647 photobleach in 10 min to a median fluorescence intensity equal to that of an unstained cell sample. **c**, Effect of photobleaching on live cells by rechallenging photobleached cells with antibodies targeting co-expressed markers. Cells stained with anti-CD45-KrO, anti-HLA-DR-APC and anti-CD3-PE were fully bleached, restained with anti-CD14, anti-CD20, anti-CD4 and anti-CD56, and compared to an unbleached control sample. No significant differences were observed. **d**,**e**, Panel design (**d**) and workflow schematic (**e**) of high-marker experiment. Antibodies and fluorophores used in each cycle are shown. PBMCs were initially tagged, stained (for Cycle 0), acquired and then photobleached. The cells were then restained for Cycle 1 antibodies and the process was repeated for Cycle 2.

We developed and optimized an in-solution photobleaching method that was compatible with live cells. To minimize viability loss during photobleaching, we built a device that illuminates cell samples while actively cooling to near 4 °C (Supplementary Fig. 6). Cells were suspended in wash buffer containing an additional cell-permeable antioxidant to prevent the formation of reactive oxygen species from damaging the cells. Using broadband light-emitting diodes (LED, 440–660 nm), we were able to photobleach a number of commonly used antibody-conjugated fluorophores (anti-CD45) in 3 to 25 min. A violet LED (400–420 nm) was needed to efficiently bleach violet-excitable fluorophores conjugated to anti-CD45 (Fig. 6a). After photobleaching, the fluorescence signal in the relevant channels (for example, both donor and acceptor components for tandem fluorophores such as PE-Cy7) is comparable to that of an unstained sample (Fig. 6b and Supplementary Fig. 7). Using 10 antibody-conjugated fluorophores at a time, the cell viability dropped slightly from 97.2% to 93.4% after a single bleach and further to 91.1% after two bleaches. We found that fluorophores conjugated to markers with higher antigen density generally tended to bleach more slowly, presumably because of limited local oxygen supply for bleaching. To verify that photobleaching does not change the relative expression of markers on cells and does not cause heterogeneous cell loss, we performed immunophenotyping of live human PBMC samples after photobleaching and compared these to a control. We found no appreciable differences between the percentages of CD4^+^ T cells, CD56^+^ NK cells, CD20^+^ B cells and CD14^+^ monocytes (Fig. 6c).

### 32-marker, 3-cycle measurement of PBMCs

To demonstrate the utility of multi-pass flow cytometry for high-parameter flow analysis, we designed a 3-cycle, 32-marker deep immunophenotyping panel of human PBMCs using 10–13 fluorophores per cycle, with photobleaching between measurements, all acquired on our 13-colour instrument (Fig. 6d). The 32 markers were chosen to enable identification of a variety of cell types including CD4^+^ T, CD8^+^ T, regulatory T, γδ T, B cells, plasmablasts, NKT-like cells, NK cells, monocytes, innate lymphoid cells and dendritic cells. For each cell type, differentiation and activation markers were included for subcategorization, such as for naïve, memory and effector T cells. Live human PBMCs were used to acquire the data. The complete cyclic workflow included LP tagging with PEI-LPs, staining with Cycle 0, acquisition, photobleaching, restaining with Cycle 1, acquisition, photobleaching, restaining with Cycle 2 and a final acquisition (Fig. 6e). In this study, ~50% of the barcoded cells that were acquired in Cycle 2 were successfully matched with previous cycles.

**Fig. 7 |:**
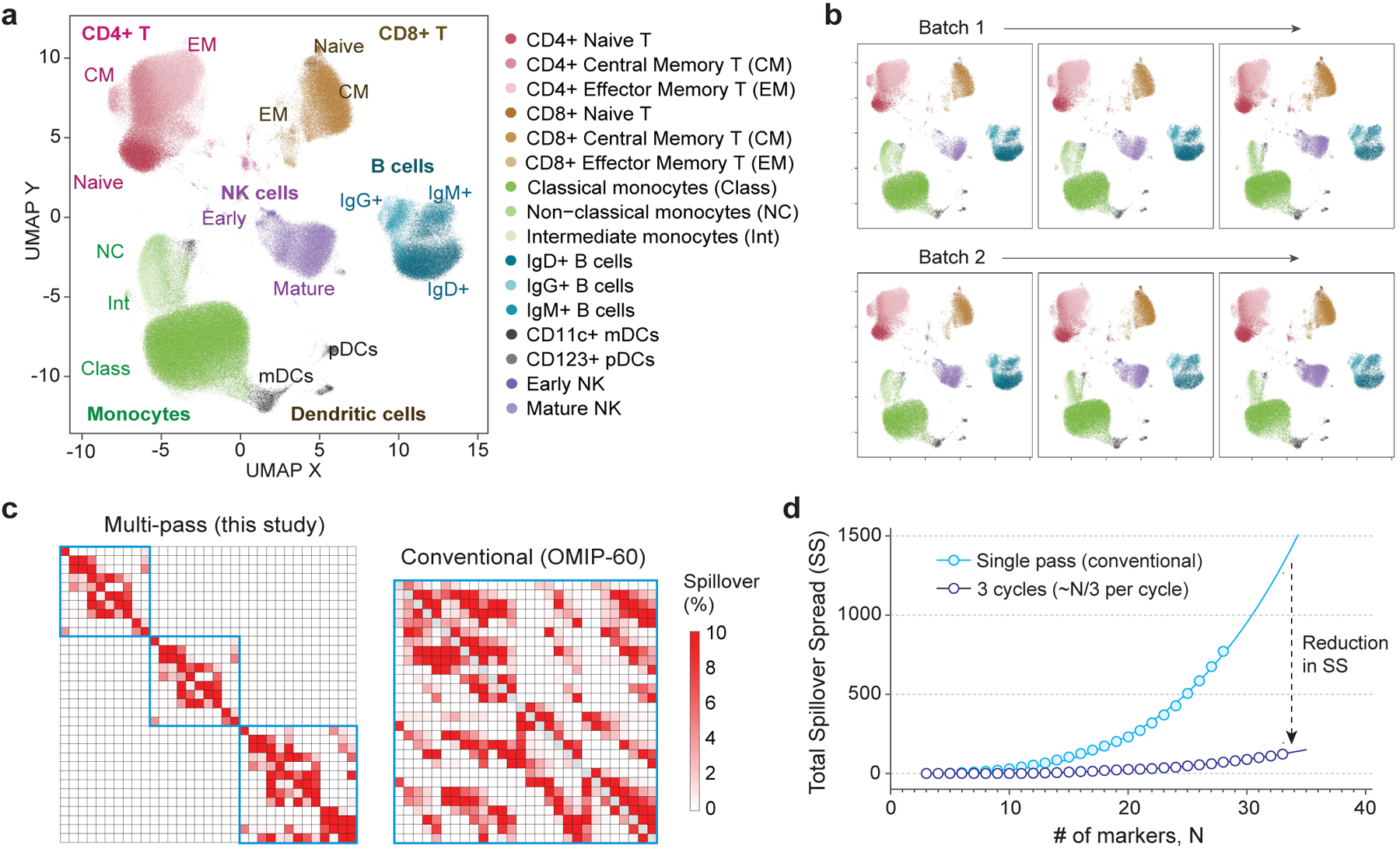
32-marker deep immunophenotyping of human PBMCs over 3 cycles. **a**, UMAP representation of data after being combined, matched and cleaned using doublet discrimination, live/dead gating and tight gating. Cell populations were manually gated and displayed on the UMAP by colour. **b**, UMAP representation of sample replicates across two batches measured on different days. No significant differences were observed with these datasets. **c**, Compensation matrix of the 32-marker, 3-cycle panel used compared to conventional 28-marker panel in OMIP-060 (ref. 40) (see Supplementary Fig. 11 for SS matrices). **d**, Simulated total SS computed for a high-marker panel using a multi-pass 3-cycle vs a conventional single-pass workflow.

To demonstrate reproducibility of our approach, we performed the assay on live human PBMCs from three healthy donors, with at least three replicates per donor. Figure 7a visualizes the matched 32-marker data from one of the donors after dimension reduction using uniform manifold approximation and projection (UMAP). At least 6 distinct islands corresponding to different cell types were observed, with good separation consistent with high data quality. Cross-referencing the UMAP pattern with each measured marker yielded the expected major cell subsets in each island, including CD4^+^ T cells, CD8^+^ T cells, CD14^+^ monocytes, CD11c^+^ dendritic cells, CD123^+^ dendritic cells, CD20^+^ B cells and CD56^+^ NK cells. We found similar results with all six replicates of this donor (Fig. 7b). Cell subpopulations were identified with <30% c.v. when frequencies were >1% for all the donors (Supplementary Fig. 8), in the acceptable range for cell-based flow assays, particularly for previously frozen PBMC samples^33–35^. Further optimization and batch processing of freeze–thawing, staining and washing steps will probably reduce this variability further. To assess whether the cyclic workflow had any effect on the final results, we also swapped the order of 10 antibodies between Cycles 1 and 2 (Supplementary Fig. 9). There were no notable differences in either the number of cell subsets identified or the quality of the data. The live-cell fraction measured in Cycle 2 for all replicates was found to range between 70 and 85%, depending on the starting viability of the PBMCs after thawing. Plotting cell viability measured at Cycle 0 vs Cycle 2 enables both identification of cells that have died and verification of the matching algorithm (Supplementary Fig 10).

We computed the compensation matrix used in our study (32 markers over 3 cycles) and compared it to a 28-marker panel optimized for conventional single-pass flow cytometry^40^. As expected, there was substantial reduction in spillover resulting from acquiring fewer colours over multiple passes (Fig. 7c). There were 136/1,056 (13%) pairs with spillover >0.5% in our panel vs 393/756 (52%) pairs in the conventional panel. When comparing panel performance within a given instrument, spillover spread (SS) is typically used to indicate how well co-expressing markers can be resolved when stained with a specific combination of colours^41,42^. We found that the total amount of SS in high-parameter panels increased nonlinearly (power law with an exponent of ~3) with each additional colour used (Supplementary Note 1). In contrast, a 3-cycle workflow had over one order of magnitude lower SS (Fig. 7d and Supplementary Note 1). Supplementary Fig. 11 shows the SS matrices of the 32-marker, 3-cycle panel from this study and the published 28-colour panel^40^.

**Fig. 8 |:**
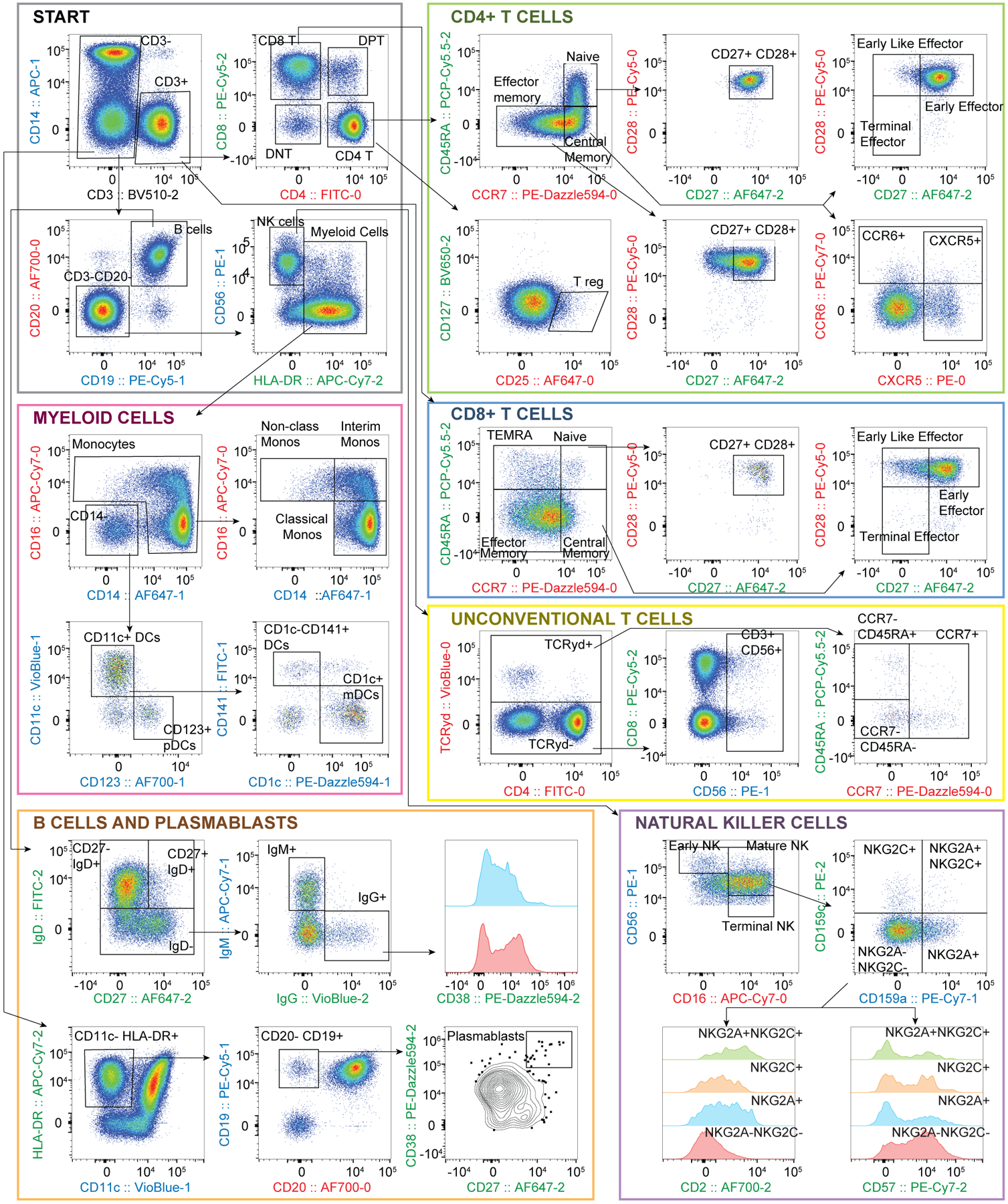
Manual gating of 32-marker, 3-cycle data. After excluding doublets and dead cells, populations of CD4^+^ and CD8^+^ T cells, unconventional T cells, myeloid cells, B cells, plasmablasts and natural killer cells were identified via manual gating. Each major cell type was further differentiated into unique subsets using markers characteristically expressed on cells from healthy human populations. Axes labels are colour coded for the cycle in which the marker was measured: red, Cycle 0; blue, Cycle 1; green, Cycle 2. Population frequencies are reported for different donors and sample replicates in Supplementary Fig. 8.

Figure 8 shows the acquired scatterplots and our gating tree. A manual gating strategy was used to distinguish T, B, NK and myeloid cell subsets, following guidelines from previously published datasets^43–47^. Briefly, T cells and their memory subtypes were identified by surface expression of CD45RA, CCR7, CD27 and CD28 on CD3^+^CD4^+^ cells or CD3^+^CD8^+^ cells. CD4^+^ helper T-cell subtypes were further differentiated by expression of CXCR5 and CCR6, and T regulatory cells were defined as CD127^lo^CD25^+^. We characterized unconventional T cells by expression of the TCRγδ or CD56 on CD3^+^ cells. Monocytes were defined as CD3^−^CD19^−^CD20^−^CD56^−^HLA^−^DR^+^ cells that expressed CD14 and/or CD16, while dendritic cells were gated with the same lineage but were here defined as lacking expression of CD14 and/or CD16. They were further characterized by expression of CD123 (pDCs) or CD1c (mDCs). Of note, this strategy may miss small subsets of dendritic cells which co-express CD16. B cells were initially defined by expression of CD19 and CD20, and lack of CD3. We used expression of IgD, IgM and IgG to differentiate B cells producing antibodies of different isotypes. Plasmablasts were identified by expression of CD19, lack of CD20 and co-expression of CD27 and CD38. Using a combination of CD16, CD56, NKG2A and NKG2C, we identified early, mature and terminal NK cells. Our results are consistent with previously published findings on T cells^44^, B Cells^45^, T_reg_ and myeloid cells^46^, as well as NK cells^47^. Importantly, we validated expected co-expression of markers by staining them in different cycles. For example, >95% of naïve CD4^+^ and CD8^+^ T cells that we have defined by CD45RA (Cycle 2) and CCR7 (Cycle 0) also expressed CD27 (Cycle 2) and CD28 (Cycle 0), as anticipated. In addition, all CD20^+^ (Cycle 0) B cells co-expressed CD19 (Cycle 1), consistent with expected healthy human phenotypes.

## Discussion

Over the past decade, the prevailing approach to improve high-marker analysis with flow cytometry has been to add more excitation lasers and detectors to the instrument while developing newer fluorophores with dissimilar properties in optical absorption and/or emission. Current high-colour commercial instruments (for example, BD FACSymphony A5 and Cytek Aurora) use as many as 10 excitation lasers and 30–188 detectors to discriminate >30 different fluorophores at a time. However, this approach comes with considerable cost and compromise. The spectral widths of organic fluorophores are typically 40–100 nm, and the detectable visible spectrum ranges from 400 nm to 800 nm. As a result, it is relatively routine to resolve up to ~10–15 different fluorophores, but beyond that, the assay difficulty increases nonlinearly for every fluorophore to be detected due to increasing SS (Supplementary Note 1). Recently introduced spectral detection can help distinguish similar colours, but spectral unmixing cannot compensate for photon shot noise and still leaves data spread^48,49^. In practice, overlapping fluorophores are not suitable for detecting co-expressing markers (Supplementary Fig. 12a). For general users, flow cytometry panels with <5 colours are considered routine, 5–10 colours are medium complexity and 10–20 colours are challenging. Panels with >20 colours are very sophisticated and can take several months to develop and optimize^9–15,50^. The time, technical expertise, reagent limitations and cost needed for current high-marker panel designs have prevented many users from increasing the number of markers that are routinely measured.

Multi-pass flow cytometry based on optical barcoding alleviates this major bottleneck to high-marker analysis. First, it simplifies a highly complex panel into multiple, easier measurements, enabling more markers to be measured with fewer colours. This reduces the time and expertise needed to optimize a high-colour panel and increases the margin of error afforded for the average user to acquire high-quality data. Second, when no more than 10–15 easily distinguishable fluorophores are used in each measurement, there is no need for expensive, custom antibody reagents conjugated with exotic fluorophores of limited availability. Widely available and well-validated antibodies with common fluorophores can be used exclusively, considerably cutting reagent costs, lead times and additional experiments needed for antibody validation. In addition, fluorophores that are the most widely available (for example, PE, APC and FITC) can be re-used multiple times. Third, the reduced number of colour channels simplifies the instrument design, instrument cost and operational complexity. Finally, by overcoming the fundamental limitation of SS when measuring many colours at once, multi-pass cytometry can exceed the maximum number of markers measured on a flow cytometer (currently, ~40 markers^43^), accelerating immunology and immune-oncology research by enabling analysis of more cell types at once.

Splitting panels across three cycles can substantially improve data quality. A conventional 30-colour experiment requires monitoring of 30 detection channels at a time and optimizing 30 × (30 − 1) = 870 spillover matrix elements for compensation. Increased spillover inevitably causes increased SS, and high-parameter panels must be carefully designed to mitigate spreading error between co-expressing antigens. In comparison, a 3-cycle × 10-colour experiment requires monitoring of 10 detection channels at a time and optimization of 3 × 10 × (10 − 1) = 270 spillover matrix elements, with most of these elements having relatively low spillover since less colours are used at a time. This dramatic overall reduction of spillover and corresponding spread contributes to major improvements of data quality (Supplementary Fig. 12b). At the same time, investigators can also design panels such that co-expressing markers are split between cycles, thereby eliminating spread between these antigens altogether. When panels are split and acquired over several cycles, even markers conjugated to the same fluorophore multiple times will not require compensating and exhibit no spillover spread between them. In addition, splitting panels between cycles allows for re-use of key fluorophores. For example, PE is a very bright and widely available fluorophore which could be used multiple times to detect low-antigen density markers. This overcomes a major limitation in conventional flow cytometry, where there is an insufficient number of bright fluorophores to detect multiple dim antigens simultaneously.

Measuring tagged cells over multiple cycles inevitably introduces some reduction to cell yield. Any step with centrifugation typically results in at least 5% cell loss, which includes cell staining and collection steps. Integration with non-centrifuging cell washing methods such as acoustic focusing^51^ or laminar flow^52^ may decrease this loss or at least reduce hands-on time and operator variability. Cell tagging yield was ~70% for live human PBMCs, which is generally limited by the Poisson statistics of the stochastic binding events. Further optimization of LP bioconjugation chemistry may improve yield and applicability to other cell types. One area of potential improvement is matching yield, which in this study was ~70% per cycle. This yield is currently limited by the presence of free LPs that contributes to matching uncertainty, cells that are excluded because they are part of cell–cell doublets in at least one cycle, and LPs that may not be detected and/or become dislodged from the cell (see Methods). Optimization of the matching algorithm to include scatter and fluorescence data is also likely to improve matching yield.

Total analysis time is increased with the cyclic workflow due to the additional steps of LP tagging, cell capture, photobleaching and restaining, apart from intentional time delay between measurements in time-lapse workflows. Currently, ~1 h is required for each additional cycle; however, only 10 min of this is hands-on time, and use of automated liquid handlers can shorten the current cell-processing time between cycles. LP tagging time and photobleaching time could be reduced further by using antibody-targeting and spectrum-optimized LEDs, respectively. Of note, LP-tagged samples can be fixed, stored and measured the next day or whenever necessary, which may be a preferred workflow for panels with intracellular markers that require fixation. Fixed samples can also be stored for batch analysis to reduce variability between specimens in large-scale clinical trials^53^. Re-interrogation of samples could also be useful for users who wish to use the results of a first cycle of measurement to inform the panel design of a subsequent measurement. The multi-pass workflow also requires that cells are run through the instrument multiple times, which imparts additional stress to the cells. However, marker expression should be minimally affected at the relatively low pressure (<3 psi) and low energy dissipation rate (~10^5^ W m^−3^) of our flow system^54–56^, and particularly sensitive markers can be deliberately acquired in the initial cycle.

The multi-pass workflow can also be leveraged in assays that require protocols or treatments that compromise fluorophore integrity. Methanol-based fixation, often used to measure the phosphorylation state of intracellular proteins, can quench protein-based fluorophores, rendering them unusable if staining precedes fixation^57^. Permeabilization buffers used to access intranuclear transcription factors often destroy signals from green fluorescent protein and other fluorescent proteins^58^. In each of these cases, phenotyping cells in an initial cycle, followed by fixation/permeabilization and subsequent measurement of intracellular markers enable measurement of all desired parameters without any compromise in signal or data quality.

## Outlook

Cellular barcoding and the multi-pass workflow expand the utility of flow cytometry beyond static profiling to the dynamic time-resolved analysis of cells at high throughput. The ability to track and measure cells over time enables the study of single-cell responses to stimulation, drug treatments or other interventions. As a one-time measurement, conventional flow cytometry can only capture cell properties at a single timepoint or assess population shifts between control and treated samples. With time-resolved flow cytometry, the downregulation or upregulation of key biomarkers on individual cells can be identified and also quantified. The degree of change in the expression of a particular biomarker on a particular cell could be especially useful for precision-medicine applications, where upregulated markers could be therapeutic targets and downregulated markers could be indicators of resistance. While PMA/ionomycin stimulation of T cells was used in this study, future applications of time-lapse cytometry could involve studying the time-dependent effects of various drugs in the development of cell-based therapies, which may lead to better predictors of efficacy. Tracking cells over multiple generations also enables the study of protein expression changes as each cell divides or differentiates, with applications in tumorigenesis and stem-cell biology.

The LPs have excitation and emission in the NIR-II range (1,000–1,700 nm), which do not interfere with existing cellular characterization techniques that rely on visible and NIR-I fluorescence. In this NIR-II window, there is less absorption and scattering by cells and tissues, which makes it suitable for live-cell and tissue applications. However, a current limitation is the need for an integrated custom NIR-II spectrometer, although LP-reading commercial instruments are under development. We anticipate that further LP-barcoding innovation and instrumentation will enable us to couple flow cytometry with other optical instruments such as a fluorescence microscope^59^. The upstream or downstream integration of spatial and functional information of single cells through LP barcoding, as illustrated in Fig. 1, promises to extend the ability to analyse single cells far beyond the current scope of flow cytometry.

## Methods

### LP fabrication

LPs were fabricated beginning from a III–V semiconductor wafer consisting of epitaxial layers of alternating InGaAsP and InP grown on an InP substrate, as previously described^20^. Nanofabrication including photolithography and reactive ion etching to create arrays of ~1.6–1.9 μm discs on the wafer were performed. The 4-inch wafer was then diced into multiple ~1 cm^2^ pieces and washed with acetone, isopropanol and deionized (DI) water. Wet chemical etching was performed by addition of 8.7 M hydrochloric acid (HCl) for 1 min to dissolve the InP layers, releasing the individual LPs. The reaction was then stopped by neutralizing the HCl with an equal amount of 8.7 M ammonium hydroxide. The etched pieces of wafer were removed and the LP solution was centrifuged at 4,000 *g* for 10 min to pellet the LPs. The LPs were then washed via 4,000 *g* centrifugation several times in pure DI H_2_O, a mixture of 1:1 DI H_2_O to ethanol (EtOH) and finally pure EtOH. Between each wash, the LPs were redispersed using an ultrasonic bath.

After washing, LPs were coated with a SiO_2_ layer to provide optical stability, enable biocompatibility, as well as to provide an appropriate surface for further functionalization. The SiO_2_ layer was formed using a modified Stöber process involving 40 mM tetraethoxysilane (TEOS) diluted in 4:1 ethanol:DI H_2_O with ammonium hydroxide as a catalyst. The LPs were reacted in the TEOS solution for 48 h on a thermomixer (ThermoMixer C, Eppendorf) at 70 °C, mixing at 1,000 r.p.m. After silica coating, the LPs were centrifuged at 4000 *g* for 8 min to remove the TEOS supernatant. LPs were washed in ethanol, 1% HCl and ethanol again via centrifugation with sonication between each wash. Silica-coated LPs were kept in ethanol until further functionalization. For functionalization with PEI polymer, the silica-coated LPs were first reacted with 3-chloropropyl triethoxysilane (CPTES) diluted in 10:1 ethanol:DI H_2_O overnight using the same thermomixer conditions as the base silica coating. Once the CPTES reaction was complete, the LPs were washed three times using pure ethanol. Next, the LPs were reacted with PEI (~1,800 Da) diluted 1:50 in DI H_2_O. LPs were then reacted with the PEI solution for 5 h in a sonicator bath using 80 kHz frequency at r.t. After the reaction, the LPs were washed using pure ethanol 3 times. For coating with biotin for antibody-based cell tagging, the base silica-coated LPs were reacted in 4 ml of 95% (v/v) EtOH with a silane-PEG-biotin, using a PEG length of 2,000 Da for 18 h at 40 °C on the thermomixer. After coating with the biotin linker, the LPs were washed 3 times with 95% (v/v) EtOH and resuspended in H_2_O at a concentration of ~100 million LPs in 1 ml of H_2_O.

### Antibody reagents

All antibody reagents are provided in Supplementary Information and are categorized by the figure they appear in.

### Multi-pass flow cytometer

A multi-pass flow cytometer was built on a commercial flow cytometer (CytoFLEX S, Beckman Coulter) by adding functionality for reading LPs as well as for collecting all cells post analysis. A nanosecond-pulsed 1,064 nm laser (Lumibird) for optically pumping LPs was integrated into the existing optical layout using an appropriate dichroic mirror and coupling optics. Several existing optical components were also replaced to ensure compatibility with LP emission in the short-wave infrared. The LP emission was collected via multimode fibres to a near-infrared grating spectrometer coupled with a fast InGaAs line-scan camera (Sensors Unlimited). For synchronized data acquisition, the line-scan camera of the spectrometer was triggered using a scattering signal from flowing cells. To collect cells after acquisition, the cells in the outlet was rerouted. A peristaltic pump at the collection end was used to draw the cells from the sample stream into a needle, reducing dilution from the surrounding sheath fluid. The normal sheath fluid was replaced with an isotonic saline solution with an added 1% (v/v) 2-phenoxyethanol surfactant. Synchronized scattering, fluorescence and LP emission were typically acquired at ~5,000 events per second at a 30 μl min^−1^ sample input flow rate.

### Multi-pass workflow experiments

To barcode PBMCs with PEI-LPs, cryopreserved PBMC samples from healthy donors were first thawed and counted. PEI-LPs dispersed in DI water at 100 MM ml^−1^ were added at a 12.5:1 ratio in serum-free RPMI-1640 medium containing 0.1 mg ml^−1^ DNase I (STEMCELL). 10X PBS was used to maintain the tonicity of the tagging solution with the addition of PEI-LPs in water at the time of tagging. The cells were tagged via centrifugation and mixing, that is, immediately after PEI-LP addition, the samples were centrifuged at 300 *g* for 5 min. Next, samples were resuspended by pipetting and mixed at 650 r.p.m. at 4 °C on a thermomixer for 5 min. The samples went through two more cycles of centrifugation and mixing and one final centrifugation. For tagging through antibody coupling, cells were first stained with 0.5 μg of anti-β2M-biotin (an antigen expressed on all nucleated cells), washed and then stained with an avidin conjugate (traptavidin, Kerafast) and washed again. Biotin-coated LPs were then added to the cells as above to tag cells via biotin–avidin binding.

To measure the effect of LP tagging and time on cell viability, one tagged sample and one control sample were immediately stained with a pre-titrated antibody cocktail (anti-CD45 Pacific Blue, anti-CD3 PE, anti-CD14 APC, anti-CD20 FITC and Zombie Aqua viability dye in PBS) and analysed on the cyclic flow cytometer. The two remaining samples were kept in 10% FBS (v/v) supplemented RPMI culture medium at 4 °C and analysed with the same antibody cocktail after 5 h or overnight incubation.

To test the effect of cell capture on cell viability, three samples of freshly thawed human PBMCs were analysed through the cyclic flow cytometer during the first cycle and post cell capture after the first and second cycles for a total of three data points per sample. Viability was determined by staining the cells with LIVE/DEAD Fixable Near-IR stain before each run. All viability measurements were compared to a non-captured control.

### Time-lapse measurements

Healthy donor human PBMCs were thawed and rested overnight in RPMI-1640 cell culture medium containing 10% (v/v) FBS and 1% (v/v) penicillin/streptomycin (P/S) at 37 °C with 5% CO_2_. The following morning, the cells were tagged with biotin-coated LPs (as described previously) and stained with a panel of releasable anti-CD45-PerCP, anti-CD3-FITC, anti-CD4-VioBlue and anti-CD8-VioGreen antibodies (Miltenyi). Samples were run on the cyclic flow cytometer, collected and then resuspended in 1% (v/v) of REAlease reagent (Miltenyi) for 10 min at r.t. to remove the Cycle 0 (C0) antibody panel. Next, the samples were stimulated and immunophenotyped. For stimulated samples, 2 μl of 1X Cell Stimulation Cocktail (eBioscience) and 1 μl of 1X Brefeldin A (BioLegend) were added per ml of cell culture medium. The samples were incubated at 37 °C and 5% CO_2_ for 4 h. Afterwards, 2 mM of EDTA was added to each sample, followed by incubation for 15 min to mitigate cell clumping. The cells were retrieved and washed. All samples were stained with LIVE/DEAD Fixable Near-IR stain in PBS with anti-CD45-FITC, anti-CD3-BV421, anti-CD4-SparkViolet 538, anti-CD8-PE/Cy5 and anti-CD14-BV605 (BioLegend). After staining, the samples were washed and fixed for 45 min at r.t. in the dark with 1X Fixation/Permeabilization Concentrate (eBioscience). The samples were washed twice with 1X permeabilization buffer (eBioscience) and resuspended in 100 μl of 1X permeabilization buffer. Cells were stained with intracellular antibodies IFNγ-PE and TNFα-APC, and then washed twice with 1X permeabilization buffer. Untagged samples were also prepared as controls. Finally, all samples were acquired by the cyclic flow cytometer.

### 10-marker, 2-cycle assay

Human T cells were isolated from freshly thawed, healthy donor PBMCs using a magnetic CD3 T-cell isolation kit (BioLegend) following manufacturer instructions. Three replicate samples were tagged with PEI-coated LPs as previously described. Samples were resuspended in 80 μl of wash buffer and stained with anti-CCR7 BV421 for 25 min at r.t., then in the same tube, added with anti-CD45RA FITC, anti-CD4 PerCP, anti-CD3 PE and anti-CD25 APC REAlease releasable antibodies (Miltenyi Biotec) for 10 min at 4 °C. The samples were washed with 2 ml of wash buffer, resuspended, acquired and captured on the cyclic flow cytometer. Next, the samples were resuspended in 1 ml of wash buffer each with 20 μl of Release reagent (Miltenyi Biotec) to release the CD45, CD3, CD4 and CD25 over a 10 min incubation at r.t. After washing again, the samples were stained with a LIVE/DEAD Fixable Green (Invitrogen) solution in 1X PBS for 5 min at r.t., then moved to 4 °C and stained with anti-CD8 PerCP, anti-CD127 PE and anti-CD27 APC for 25 min. All three replicate samples were washed before acquiring on the cyclic flow cytometer.

### Photobleacher

Two in-house LED-based fluorochrome photobleachers were designed and fabricated. A high-power, 3,500-K white LED (Luminus Devices) and 100 W, 405 nm LED (Chanzon) were used to illuminate samples to deactivate antibody-conjugated fluorophores. A chiller module to pump ice water to the sample holder kept the sample cool during photobleaching to ensure cell viability.

### Photobleaching experiments

Fourteen common fluorophores were characterized for photobleaching as part of the cyclic flow cytometry workflow. Anti-CD45 antibodies conjugated to the 14 fluorophores were used to stain freshly thawed human PBMCs (AllCells). Cells (10^6^) were photobleached in 2 ml of ‘wash buffer’, which contains 10% (v/v) fetal bovine serum, 0.1% non-ionic surfactant, 2 mM EDTA and 10 mM HEPES in 1X PBS without calcium or magnesium. A cell-permeable form of vitamin E (Trolox, VectaCell) was also added to the buffer as an antioxidant to protect cells from potential free-radical damage. Median fluorescence intensities (MFIs) were compared against an unstained control every ~1–5 min of sample illumination to determine the minimum necessary exposure time to erase the CD45^+^ fluorescent signals (see Fig. 6a).

To test the effect of photobleaching on immunophenotyping, PBMCs were labelled and measured with an antibody panel ‘panel A’ (anti-CD3 PE, anti-HLA-DR-APC and anti-CD45 KrO), panel A was photobleached and the PBMCs were restained with a subsequent antibody panel ‘panel B’ (anti-CD4 PE, anti-CD20 FITC, anti-CD14 APC and anti-CD56 BV421). MFIs were measured against control samples stained with panel B that did not undergo photobleaching. Samples stained with panel A were photobleached for 30 min. Fluorescence signal erasure was verified by flow cytometry. Photobleached samples were collected, washed and restained with panel B, acquired and compared to the control.

To test the effect of photobleaching on cell viability, four samples of freshly thawed human PBMCs were stained with panel C0, photobleached completely (15 min) in wash buffer and restained with either LIVE/DEAD Fixable Near-IR stain (Life Technologies) or panel C1. The two samples restained with panel C1 were photobleached and the samples were then restained with LIVE/DEAD Fixable Near-IR stain. All viability measurements were compared to the non-photobleached control.

### 32-marker 3-cycle experiment

Freshly thawed PBMCs from three healthy donors (AllCells, Zen-Bio) were counted and tagged with PEI-coated LPs as described previously. Each tagged sample replicate (at least 3 per donor) was resuspended in 80 μl of (2:500) LIVE/DEAD Fixable Near-IR solution in 1X PBS. Samples were first stained with the titrated C0 antibody cocktail for 25 min at r.t. in the dark. Once stained, samples were washed with 3 ml wash buffer and centrifuged for 5 min at 300 *g*. All samples were resuspended to ~100 μl in wash buffer, analysed on the cyclic flow cytometer and collected. Samples were transferred promptly to the photobleacher, in which fluorophores were bleached for 15 min. The photobleached samples were diluted with wash buffer, washed and resuspended in 80 μl of LIVE/DEAD Fixable Near-IR solution in 1X PBS. Samples were then stained with the subsequent C1 antibody cocktail for 25 min at 4 °C, and the entire process was repeated to acquire C1 and C2 data. For repeatability tests, the above experiment was conducted on one donor in two sets of triplicates over 2 different days (Day 1 batch and Day 2 batch). To test the effect of the cyclic workflow, another experiment was conducted with a modified panel where a total of 10 antibodies corresponding to 5 fluorochromes were swapped between cycles 1 and 2 (Cycle 0, 2, 1) The swapped antibodies were anti-CD8 and anti-CD19 PE/Cy5, anti-CD159c and anti-CD56 PE, anti-CD38 and anti-CD1c PE/Dazzle594, anti-CD57 and anti-CD159a PE/Cy7, and anti-CD27 and anti-CD14 Alexa Fluor 647.

Fluorescence minus one (FMO) controls were used to identify spectral overlap that can cause false-positive signals and gating ambiguities with dim antigens. These controls were run for all donors and used to define accurate gate placement for selected markers. The first FMO included all antibodies in C0 except for anti-CD25 Alexa Fluor 647, and the second FMO included all antibodies in C0 except for anti-CCR6 PE/Cy7. We used the FMOs to dictate where we distinguished CD25^+^ from CD25^−^ and CCR6^+^ from CCR6^−^ in the fully stained replicate samples. In place of the missing antibody for each FMO control, we used an isotype control.

The spillover spread matrix (SSM) for OMIP-060 (ref. 40) was computed in FlowJo v.10.9.0 (BD) using publicly available flow cytometry standard (FCS) files hosted on FlowRepository. For the conventional panel, the contribution of each fluorophore towards overall spread was calculated by adding the spread of each fluorophore into a given detector to the spread of the detector’s corresponding fluorochrome into all other detectors. Fluorophores with the largest contributions towards spread were deleted systematically from the matrix one at a time to simulate an SSM with the least possible amount of spread as the number of fluorophores was decreased from 28 to 1. The resulting total SSM was recorded after each fluorochrome deletion. For the simulated 3-cycle panel, the combination of 11 fluorophores with the smallest SSM was identified by using the conventional SSM and systematically deleting fluorophores with the largest contributions to spread until 11 remained in the panel. This panel was replicated three times (for each cycle) to build a theoretical SSM with 33 fluorophores representing the least possible amount of spread. As with the conventional panel, total fluorophore contributions towards spread were calculated and SSM totals were recorded as the number of fluorophores was decreased one by one.

### LP matching algorithm

FCS files containing fluorescence and LP data were imported to the matching algorithm. The *m* central wavelengths and amplitudes of LP emission lines from each cell *i* were recorded as $\lambda_{i}^{\left(m\right)}$ and $A_{i}^{\left(m\right)}$ respectively, for up to the 15 most intense spectral peaks. These 2*m* parameters constitute a ‘barcode’, $\Lambda_{i}\overset{\text{def}}{=}\left\{\left(\lambda_{i}^{\left(m\right)},A_{i}^{\left(m\right)}\right)\right\}$. For two cycles of measurement, this information produces a set of barcodes $\Lambda^{1}=\left\{\Lambda_{i}^{1}\right\}$ for Cycle 1 and another set $\Lambda^{2}=\left\{\Lambda_{i}^{2}\right\}$ for Cycle 2. To compare any two barcodes Λ_*i*_ and Λ_*j*_ taken from either **Λ**^**1**^ or **Λ**^**2**^, we defined a score function

$$S\left(\Lambda_{i},\Lambda_{j},\alpha \right)=\sum_{(\text{m},\text{n})\in \text{matched}}\text{log}\left(p\left(\lambda_{i}^{\left(m\right)},A_{i}^{\left(m\right)},\lambda_{j}^{\left(n\right)},A_{j}^{\left(n\right)},\alpha \right)\right)\;+\sum_{\text{r}\in \text{not matched}}\text{log}\left(q\left(\lambda_{i}^{\left(r\right)},A_{i}^{\left(r\right)},\alpha \right)\right)\;+\sum_{\text{s}\in \text{not matched}}\text{log}\left(\text{q}\left(\lambda_{j}^{\left(s\right)},A_{j}^{\left(s\right)},\alpha \right)\right),$$

where ***α*** denotes a set of tuning parameters. The first sum included all pairs of ‘matched’ peaks (*m*, *n*) that are within 2 nm from each other. The second and third sum included ‘not matched’ peaks, that is, ones observed in one barcode but missing in the other. The tuning parameters ***α*** are chosen such that *p* ≈ 1 and *q* ≪ 1. For every barcode in Cycle 2, there is a corresponding barcode from Cycle 1 with the highest score; the set of highest scores give a normalized distribution *S*_12_. The set of highest scores between each cell and all other cells in the same cycle gives another normalized score distribution *S*_11_. Since a pair of matched barcodes generates a higher score than almost any random pairs of barcodes, there exists a population of high scores in *S*_12_, corresponding to the matched pairs, that does not exist in *S*_11_. We define a merit function acting on the two distributions, Δ*S* [*S*_12_, *S*_11_], as a quantitative measure of this difference, which approximately represents the fraction of cells from Cycle 2 that can be matched with cells from Cycle 1. We determined the tuning parameters α* that maximized the Δ*S*. Next, using these parameters, we computed the probability that cell *i* in Cycle 1 and cell *j* in Cycle 2 corresponded to the same cell: *P*_*ij*_ = *S* (Λ_*i*_, Λ_*j*_, ***α***^∗^) / ∑_*kl*_ *S* (Λ_*i*_, Λ_*k*_, ***α***^∗^) *S* (Λ_*l*_, Λ_*j*_, ***α***^∗^) where Λ_*k*_ and Λ_*l*_, denote all potential matches to cell *i* and cell *j*, respectively. Finally, pairs of cells with the maximum *P*_*ij*_ produces a set of initial ‘barcode-matched’ cells.

As quality control, we rejected matched cells with low matching probability using a cut-off value for *P*_*ij*_, which we validated through the following two control experiments. The first experiment involved staining LP-tagged PBMCs (~10^5^ cells) with multiple antibodies (CD45, CD19, CD14, CD3), acquiring the cells on the cyclic flow cytometer, collecting the cells and then acquiring again. The data were matched to assess correlation of fluorescence signals between measurements. The second experiment involved measuring three separate LP-tagged samples (each with ~10^5^ cells) over two cycles. The data from all 3 samples were pooled together and matched to track sample identity compared to ground truth. The lowest probability *P*_*ij*_ that yielded >98% correlation of fluorescent signals and >98% accuracy in identifying samples was chosen as the cut-off value.

### Flow cytometry data analysis

After LP barcode matching, data from all samples and for each cycle were concatenated into one master file in the FCS format. FCS files were analysed using FlowJo. Single-colour compensation controls were acquired with every run for all assays and typically prepared using UltraComp eBeads and ArC beads (Invitrogen) at voltages dictated by instrument voltration. Approximately 7,500 events per sample were recorded to generate compensation matrices for each cycle. Manual adjustments to compensation were applied to ensure that the MFI of single-positive populations matched the MFI for negative populations for all fluorophores for which they were negative.

For cyclic experiments, compensation matrices for each cycle were calculated. The final compensation matrix was generated by manually combining the compensation matrices from each individual cycle and setting compensation between fluorophores in different cycles to 0. Compensation controls were additionally used to calculate the SSM for each individual cycle automatically in FlowJo^41^. SSM data from all three cycles were manually combined, and the spillover spreads between fluorophores in different cycles were set to 0. The frequency of LP-tagged and LP-untagged lineage cell populations (CD3^+^ T cells, CD20^+^ B cells and CD14^+^ monocytes) were compared when applicable. Any observed differences in cell frequencies were normalized to match the frequencies of untagged and unmatched cells. Data were cleaned by using a series of tight gates that excluded scattered cells showing double positivity for multiple lineages (for example, CD3^+^CD20^+^ cells were removed) and cells that were identified as debris on the basis of a combination of fluorescence and forward-scatter/side-scatter characteristics.

The frequency, mean and %c.v. using s.d./mean were calculated for all replicates and populations that exceeded 100 cell events and >0.5% of the parent population. For the 32-marker experiment, Donor 3’s Batch 1 and Batch 2 sample population means and c.v.s were calculated both separately and combined. Compensation matrices were calculated in the FlowJo Compensation Wizard using single-colour controls (see Fig. 5 and Supplementary Figs. 5 and 8). To generate the UMAPs for the 32-marker panel, 50,000 cells were downsampled from each of the 6 replicates from Donor 3 taken across two batches and concatenated into one file. This concatenated file was used to generate a master UMAP, which was calculated on the basis of all compensated fluorescent parameters, except for live/dead (which was used in the upstream cell selection), using a Euclidean distance function with nearest neighbours =15 and minimum distance =0.5. After running the UMAP algorithm, manual gating was employed to identify specific cell populations that corresponded with UMAP clusters. Individual replicates were identified from the master file by gating on the basis of ‘Sample ID’, enabling identification of each replicate for analysis. Data from these populations and replicates were combined, imported and visualized in R using the ‘tidyverse’ package.

### Reporting summary

Further information on research design is available in the Nature Portfolio Reporting Summary linked to this article.

## Supplementary Material

Supplementary Figures

Supplementary Figure Antibody List

Main Figure Antibody List

## Data Availability

The main data supporting the results in this study are available within the paper and its Supplementary Information. Source data for the flow-cytometry plots are available in FlowRepository (experiment ID: FR-FCM-Z7ZJ). Source data are provided with this paper.
