# Supplementary material for: High-dimensional multi-pass flow cytometry via spectrally encoded cellular barcoding
Source: Nat Biomed Eng. 2023 Nov 30;8(3):310–24. doi: 10.1038/s41551-023-01144-9 (PMC10963263; doi:10.1038/s41551-023-01144-9)
Supplement: Supplementary file 1 [file 41551_2023_1144_MOESM1_ESM.pdf]

nature biomedical engineering

Article https://doi.org/10.1038/s41551-023-01144-9

High-dimensional multi-pass flow cytometry via spectrally encoded cellular barcoding

In the format provided by the
authors and unedited

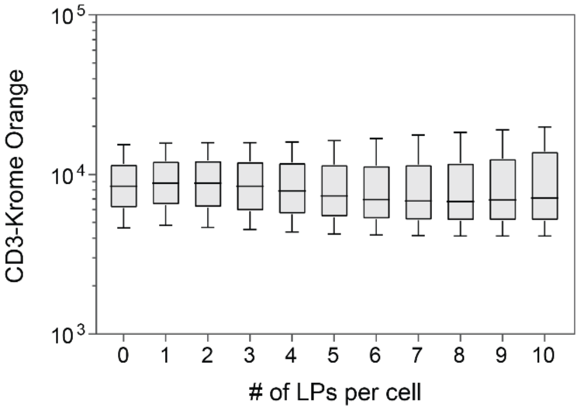

Supplementary Fig. 1 | Dependence of CD3-Krome Orange fluorescence intensity on number of LPs per cell. Box plots show line at median, with error bars spanning the 10-90 percentiles.

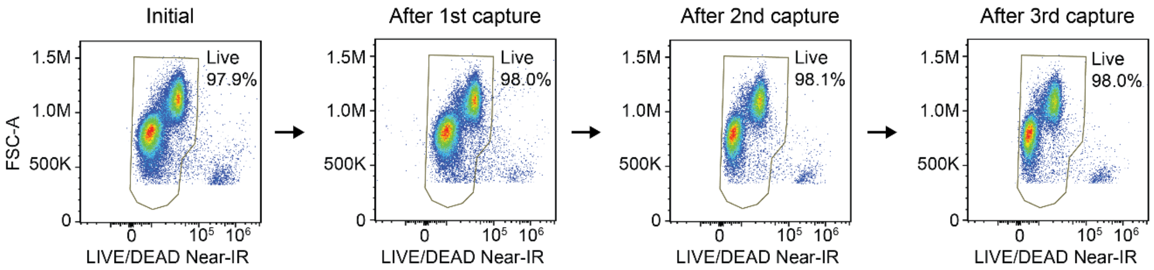

Supplementary Fig. 2 l Cell viability of human PBMCs measured after 0,1,2, and 3 successive captures. Plots depict all CD45+ singlet events.

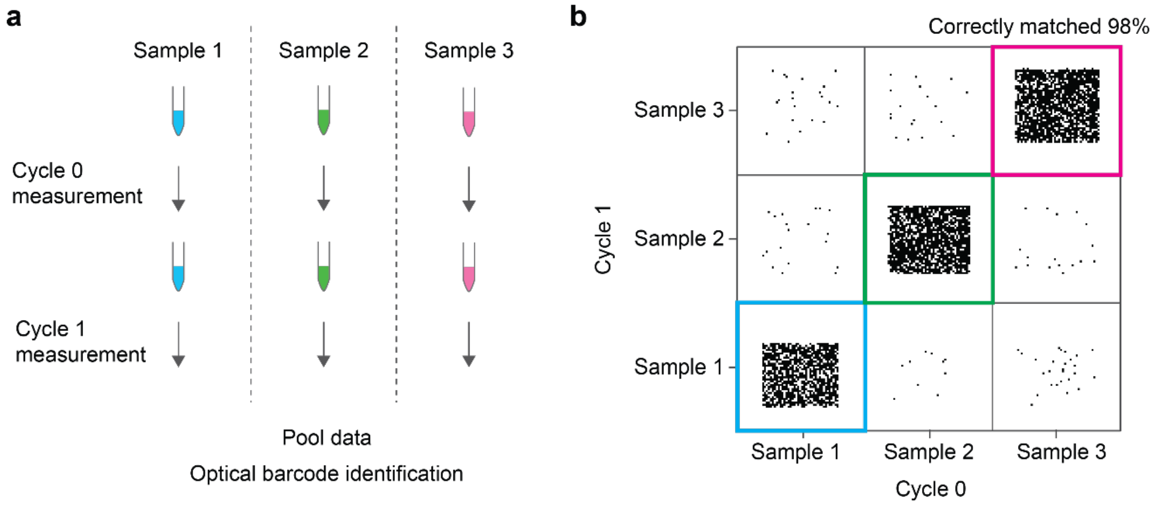

Supplementary Fig. 3 l Validation of LP barcode matching. (a) Three cell samples each with ~200,000 barcoded cells were acquired and collected separately over 2 cycles. Data from the 3 samples were concatenated and matched to assess the accuracy of matching. (b) 98% of the matched cells were correct in maintaining sample identity across cycles. Plot only displays 6,000 cells out of ~360,000 for clarity.

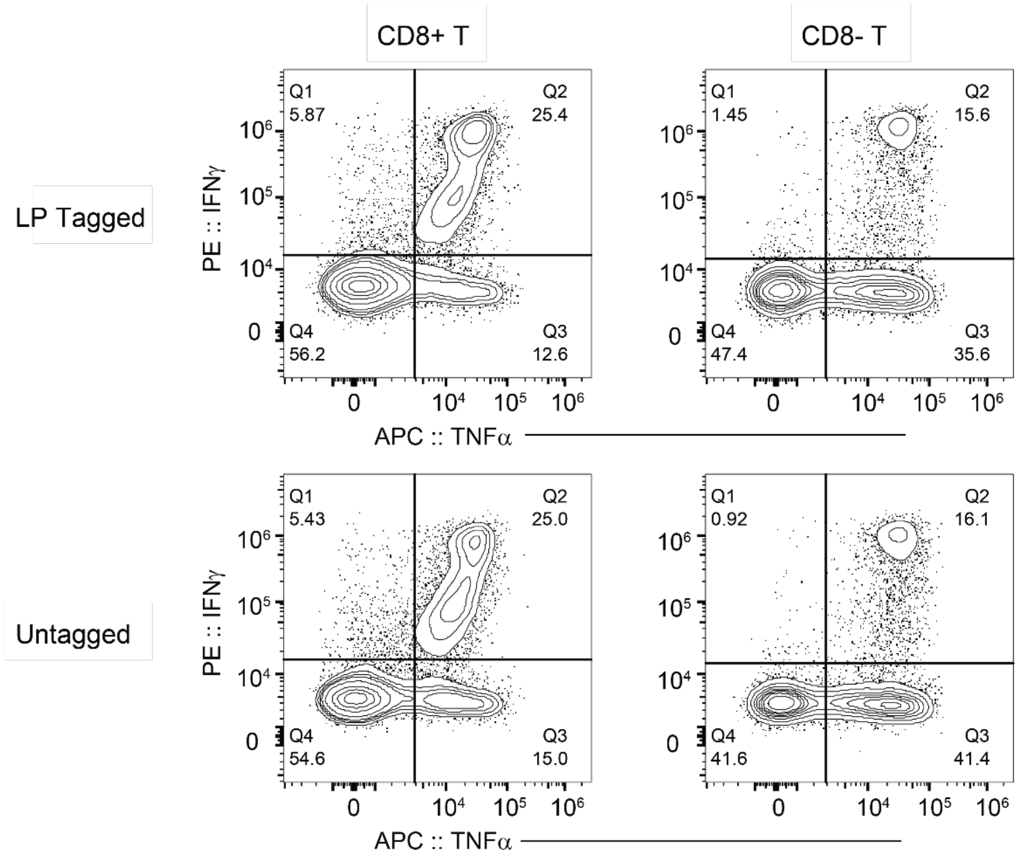

Supplementary Fig. 4 I Cytokine secretion of stimulated human T cells with and without LP tagging. No substantial differences in IFNg and TNFa secretion were observed (beyond typical batch variations of ~20% CV) between tagged and untagged cells, for both CD8+ and CD4+ T cells.

	% of parent				
Population	S1	S2	S3	Mean	% CV
Live/CD4+	76.4	75.9	72.2	74.8	3.07
CD4+/Central Memory	50.4	49.9	55.5	51.9	5.97
CD4+/Central Memory/CD27+CD127+	84.4	85.9	83.9	84.7	1.23
CD4+/Central Memory/CD27+CD127-	14.6	12.9	13.7	13.7	6.19
CD4+/Effector Memory	33	31.3	28.3	30.9	7.71
CD4+/Effector Memory/CD27+CD127+	72.9	74.3	71.6	72.9	1.85
CD4+/Effector Memory/CD27+CD127-	18.3	17.3	15.7	17.1	7.67
CD4+/Effector Memory/CD27-CD127+	7.48	7.28	10.3	8.4	20.22
CD4+/Effector Memory/CD27-CD127-	1.11	1.05	1.48	1.2	19.19
CD4+/Naive	15.1	17.2	14.6	15.6	8.82
CD4+/Naive/CD27+CD127+	74.5	77.1	71.3	74.3	3.91
CD4+/Naive/CD27+CD127-	25	22.5	26.4	24.6	8.02
CD4+/Tregs	2.55	2.72	3.4	2.9	15.56
Live/CD8+	18.2	18.8	19.9	19.0	4.55
CD8+/Central Memory	33.2	35.1	34.4	34.2	2.81
CD8+/Central Memory/CD27+CD127+	91.6	92.1	94.1	92.6	1.43
CD8+/Central Memory/CD27+CD127-	5.24	5.17	3.03	4.5	28.04
CD8+/Central Memory/CD27-CD127+	3.04	2.52	2.76	2.8	9.38
CD8+/Effector Memory	55.8	52.4	54.8	54.3	3.22
CD8+/Effector Memory/CD27+CD127+	77.4	76.8	82.5	78.9	3.97
CD8+/Effector Memory/CD27+CD127-	13.4	14.3	8.49	12.1	25.92
CD8+/Effector Memory/CD27-CD127+	5.24	5.33	6.24	5.6	9.87
CD8+/Effector Memory/CD27-CD127-	3.75	3.38	2.59	3.2	18.29
CD8+/Naive	4.78	5.12	3.35	4.4	21.27
CD8+/Naive/CD27+CD127+	86.4	85.8	89.9	87.4	2.53
CD8+/Naive/CD27+CD127-	13.4	13.8	9.55	12.3	19.16
CD8+/TEMRA	6.19	7.15	6.82	6.7	7.26
CD8+/TEMRA/CD27+CD127+	68.5	69.5	74.5	70.8	4.54
CD8+/TEMRA/CD27+CD127-	25.6	26.3	20.9	24.3	12.10
CD8+/TEMRA/CD27-CD127+	1.93	1.3	1.46	1.6	20.95
CD8+/TEMRA/CD27-CD127-	3.95	2.8	3.07	3.3	18.37

Supplementary Fig. 5 l Isolated T cells from human cryopreserved PBMC were stained with a 2-cycle, 10-marker panel of releasable antibodies. Percentages and mean of CD4+ and CD8+ memory T cell subpopulations are shown for three replicate samples (S1, S2, and S3) taken across multiple days from the same donor. Values are expressed as a percent of the parent population and colored by a heat scale with blue representing the largest values and white representing the smallest. % CV was calculated using SD/mean, where SD is the standard deviation of the samples. A red color bar representing the % CV enables visual comparison between cell populations.

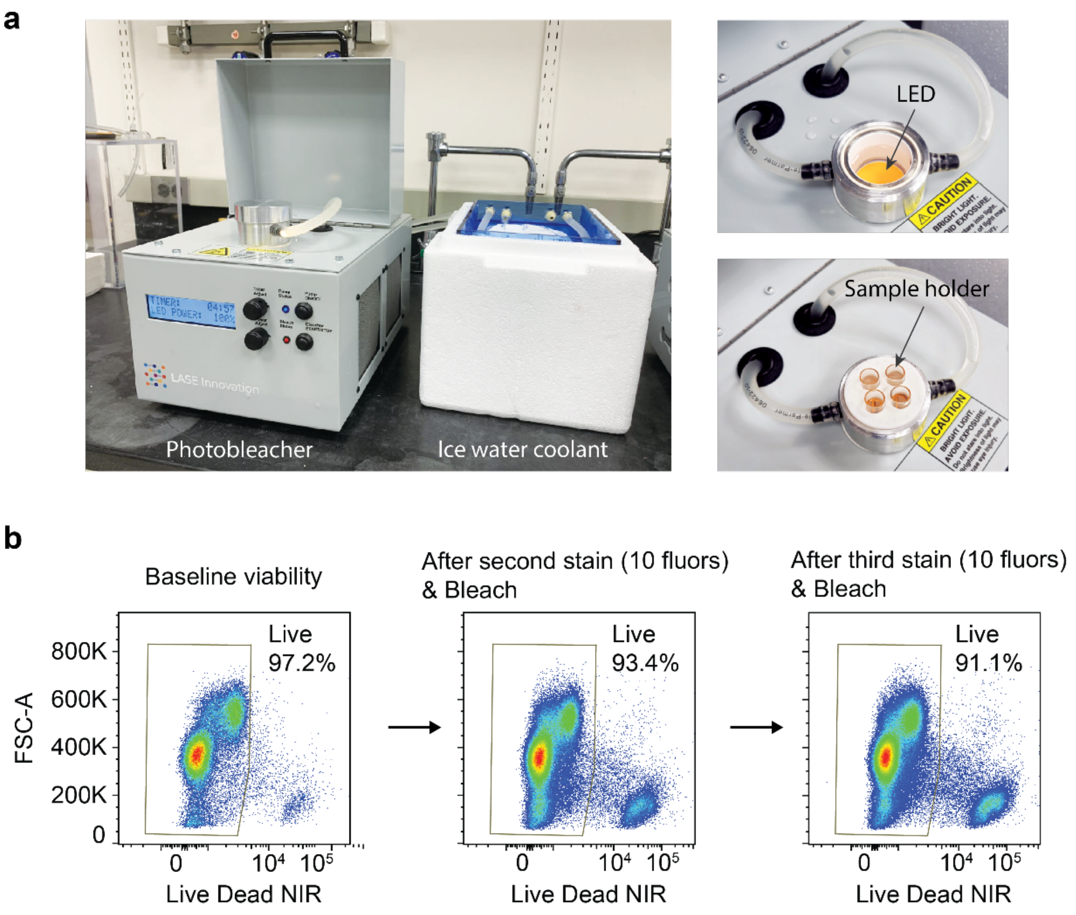

Supplementary Fig. 6 I (a) Image of custom-built photobleaching device in which up to 4 samples are photobleached using a bright LED while being cooled to close to 4°C. (b) Viability of live human PBMCs following complete photobleaching of samples stained with 10 fluorophores (Cycle 0 in Fig. 6d) and subsequently another 10 fluorophores (Cycle 1). The cell viability decreases around 4% per cycle. Plots depict all singlet events.

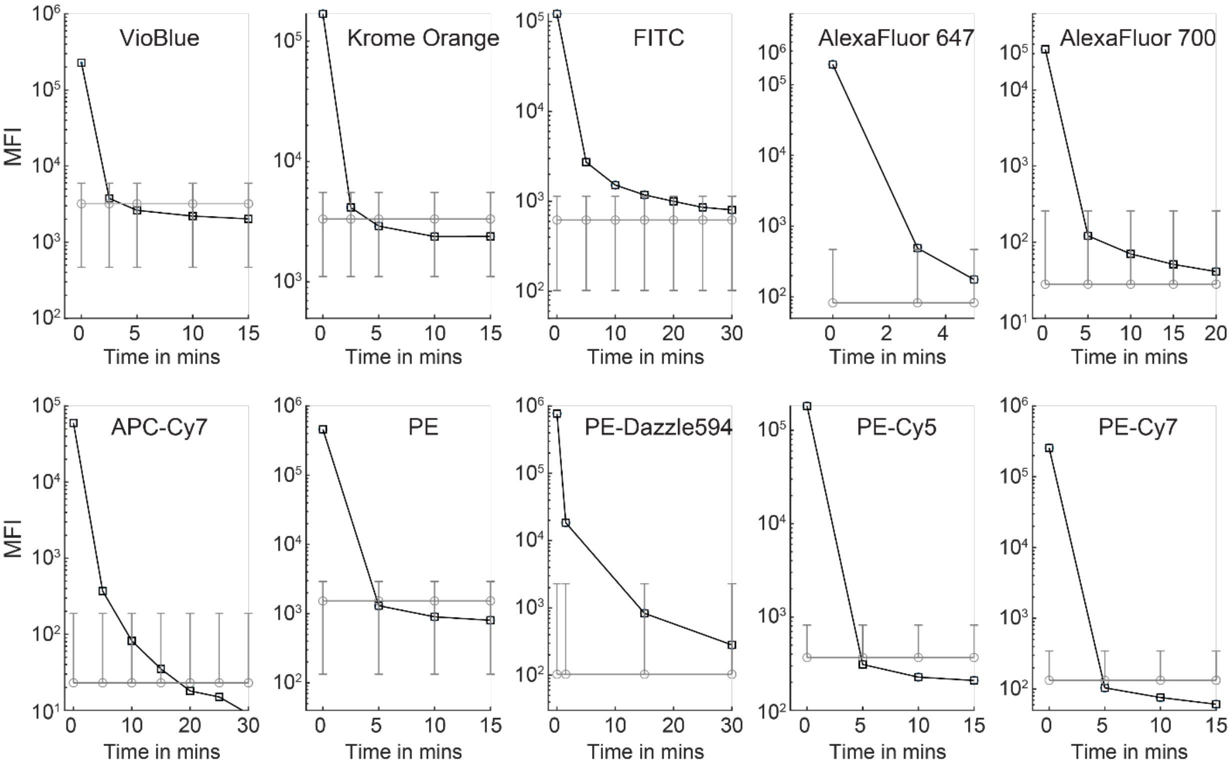

Supplementary Fig. 7 | Fluorophore photobleaching kinetics. Fluorophores conjugated to anti-CD45 were used to stain >10^6 live human PBMCs each. Every 5 min or less aliquots of >10^5 cells were removed from each sample and acquired (black) on a flow cytometer to monitor MFI. An unstained sample was also acquired (grey). Error bars indicate +/- standard deviation about the median of the unstained sample's fluorescence intensities in that respective channel (noise).

	Donor 1					Donor 2					Donor 3- Batch 1					Donor 3- Batch 2					Donor 3 Combined	
**Population**	**S1**	**S2**	**S3**	**Mean**	**CV**	**S1**	**S2**	**S3**	**Mean**	**CV**	**S1**	**S2**	**S3**	**Mean**	**CV**	**S1**	**S2**	**S3**	**Mean**	**CV**	**Mean**	**CV**
**B cells (% of total)**	8.95	8.83	8.79	8.9	1%	17.4	17.2	17.1	17.2	1%	11.5	11.6	11.6	11.6	0%	12	11.8	11.7	11.8	1%	11.7	2%
B cells/CD27-IgD+	53.5	52.9	53.1	53.2	1%	75.9	79.1	78.2	77.7	2%	62.4	61.4	60.6	61.5	1%	51.1	51.2	50.7	51.0	1%	56.2	10%
B cells/CD27+IgD+	5.43	4.63	5.33	5.1	8%	6.34	4.38	2.91	4.5	38%	5.9	4.71	3.96	4.9	20%	7.04	7.97	5.37	6.8	19%	5.8	25%
IgD-/IgG+	21.2	18.8	20.7	20.2	6%	16.3	14.7	13	14.7	11%	11	9.89	9.1	10.0	10%	9.33	8.4	8.99	8.9	5%	9.5	10%
IgD-/IgM+	20.8	20.7	19.5	20.3	4%	19.2	19.3	18.7	19.1	2%	34.7	32.5	32.4	33.2	4%	39.3	38.7	37.1	38.4	3%	35.8	8%
**Monocytes (% of total)**	51.6	51.9	51.6	51.7	0%	39.7	39.1	39.7	39.5	1%	18.2	18.6	16.6	17.8	6%	22.4	21.9	20.5	21.6	5%	19.7	12%
Monos/Classical	79.1	80	81.3	80.1	1%	97.4	98.2	97.3	97.6	1%	84.7	85.6	87	85.8	1%	89.2	89.8	89.3	89.4	0%	87.6	2%
Monos/Interm	11.3	10.9	10.3	10.8	5%	2.34	1.61	2.33	2.1	20%	14.1	13	11	12.7	12%	9.43	8.47	8.58	8.8	6%	10.8	22%
Monos/Non-class	7.82	7.42	6.85	7.4	7%	0.27	0.21	0.35	0.3	25%	1.15	1.41	1.99	1.5	28%	1.35	1.68	2.11	1.7	22%	1.6	23%
**Dendritic cells (% of total)**	5.13	6.28	5.89	5.8	10%	4.86	4.76	4.21	4.6	8%	4.97	4.96	4.44	4.8	6%	3.8	4.08	3.85	3.9	4%	4.4	12%
CD14-CD16-/CD11c+ DCs	37.7	42.9	40.3	40.3	6%	28.4	27.6	32	29.3	8%	64.7	66.3	66.4	65.8	1%	68.8	67.4	68	68.1	1%	66.9	2%
CD14-CD16-/CD123+ pDCs	47.3	42.8	44.6	44.9	5%	39.2	43.8	44.9	42.6	7%	18.1	14.6	14.2	15.6	14%	14.5	14.3	14.3	14.4		15.0	10%
**NK cells (% of total)**	11.6	12.6	13.4	12.5	7%	4.27	3.87	4.12	4.1	5%	13.9	14.6	16.3	14.9	8%	15	15.2	16.5	15.6	5%	15.3	7%
NK cells/Early NK	7.11	7.06	6.25	6.8	7%	11.7	15.9	12.4	13.3	17%	3.72	3.73	3.06	3.5	11%	3.93	3.52	2.98	3.5	14%	3.5	11%
NK cells/Mature NK	69.3	64.8	65.2	66.4	4%	45.3	43.7	44.3	44.4	2%	54.2	49.1	50.3	51.2	5%	36.5	33.3	34	34.6	5%	42.9	22%
**T cells (% of total)**	22.8	20.5	20.3	21.2	7%	33.8	35.1	34.9	34.6	2%	51.4	50.2	51.1	50.9	1%	46.9	47.2	47.6	47.2	1%	49.1	4%
CD3+/CD4+	64.1	61.1	57.7	61.0	5%	27.9	29.1	24.7	27.2	8%	68.2	68.4	69.2	68.6	1%	63.7	64	63.4	63.7	0%	66.2	4%
CD3+/CD4+/Naive	34.2	32.8	33.8	33.6	2%	20	26.1	18.6	21.6	18%	14.8	14.3	14.6	14.6	2%	16.3	16.5	16.7	16.5	1%	15.5	7%
CD4+/Effector Memory	27	28	27.5	27.5	2%	69.4	63.9	70.9	68.1	5%	45.1	47.8	45.1	46.0	3%	45.1	42.4	42.5	43.3	4%	44.7	5%
CD4+/Central Memory	38.2	38.3	37.9	38.1	1%	8.72	7.66	8.91	8.4	8%	39.7	37.5	39.8	39.0	3%	38.5	40.9	40.5	40.0	3%	39.5	3%
CD4+/CD127lo CD25+	3.37	3.46	3.11	3.3	5%	3.33	2.55	3.22	3.0	14%	1.02	1.22	0.99	1.1	12%	1.2	0.88	0.97	1.0	16%	1.0	13%
CD3+/CD8+ T	27	29.6	33.1	29.9	10%	62.7	61.6	66	63.4	4%	26.2	26	25.3	25.8	2%	30	28.9	30.6	29.8	3%	27.8	8%
CD8+ T/Central Memory	10.5	10.2	10.5	10.4	2%	3.27	2.87	3.45	3.2	9%	11.6	10.1	9.43	10.4	11%	8.74	9.94	8.48	9.1	9%	9.7	12%
CD8+ Central Memory/TE	0.59	0.58	1.31	0.8	51%	28.6	32.3	24.0	28.3	15%	0.92	0.82	1.14	1.0	17%	0.81	0.88	0.65	0.8	15%	0.9	19%
CD8+ T/Naive	31.8	32.6	33.9	32.8	3%	5.89	6.11	5.57	5.9	5%	1.26	1.15	1.24	1.2	5%	1.7	1.59	1.48	1.6	7%	1.4	16%
CD8+ T/Effector Memory	42.4	41.7	41.3	41.8	1%	59.6	66	67.8	64.5	7%	81.2	83.1	83	82.4	1%	83.2	82.8	84.2	83.4	1%	82.9	1%
CD8+ T/TEMRA	14.6	13.9	13.5	14	4%	30.1	24.3	22.6	25.7	15%	5.66	5.32	5.66	5.5	4%	5.31	4.76	4.66	4.9	7%	5.2	8%
CD3+/DN T	7.27	7.74	7.62	7.5	3%	6.81	7.13	7.15	7.0	3%	1.89	2.29	2.38	2.2	12%	2.55	3.27	2.64	2.8	14%	2.5	18%
CD3+/DP T	0.95	0.82	0.92	0.9	8%	1.8	1.67	1.6	1.7	6%	2.95	2.66	2.31	2.6	12%	3.34	3.1	2.88	3.1	7%	2.9	12%
TCRgd-/CD3+CD56+	8.1	8.1	8.32	8.2	2%	19.3	17.7	19.1	18.7	5%	2.52	2.19	2.57	2.4	9%	2.54	2.55	2.71	2.6	4%	2.5	7%
CD3/TCRgd+	3.56	3.75	3.79	3.7	3%	5.52	5.48	6.03	5.7	5%	0.6	0.93	1.08	0.9	28%	1.2	1.32	1.39	1.3	7%	1.1	27%

Supplementary Fig. 8 l Human cryopreserved PBMCs were stained with a 3-cycle, 32 marker panel of conventional fluorescent antibodies. Representative cell populations with percentages that were >0.5% of the parent and had at least 100 cells in the gate are shown for three replicate samples (S1, S2, and S2) per donor for three donors. Replicates from one donor were acquired in two independent experiments (batches). Values are expressed as a percent of the parent population and colored by a heat scale with blue representing the largest values and white representing the smallest. % CV was calculated using SD/mean, where SD is the standard deviation of the samples. A red color bar representing the % CV enables visual comparison between cell populations.

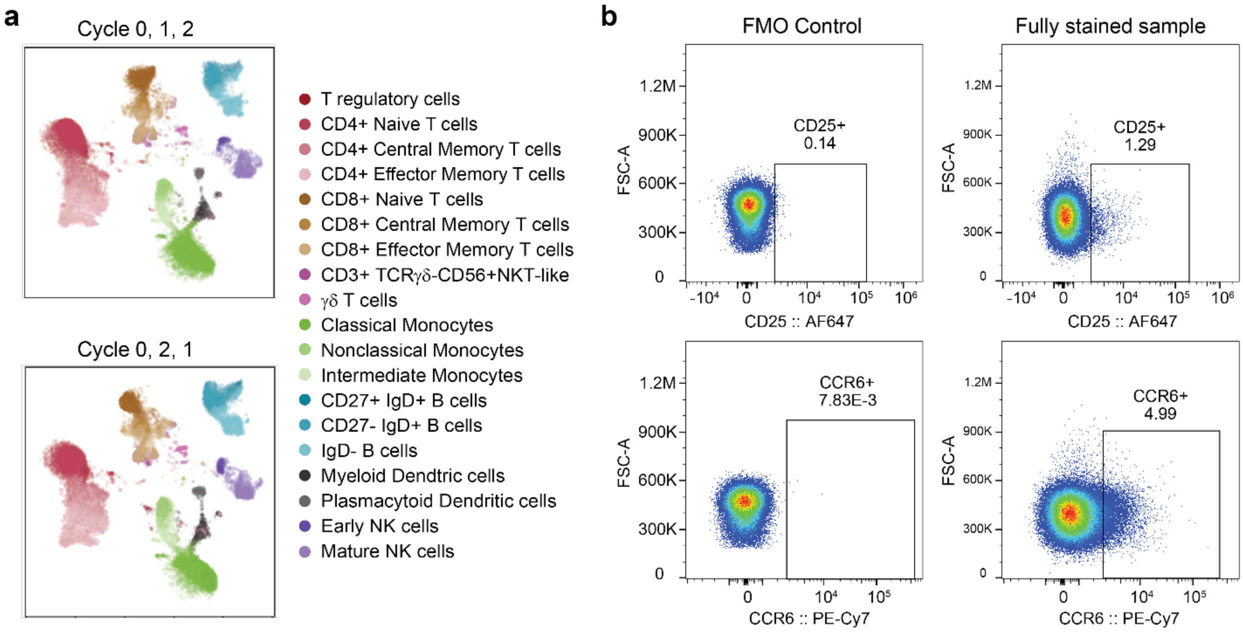

Supplementary Fig. 9 l Experimental controls for 3-cycle, 32-marker deep immunophenotyping panel. (a) Top: UMAP analysis depicts cell populations identified using the original panel design (Cycle 0, Cycle 1, then Cycle 2). Bottom: UMAP analysis of a second iteration where the order of the second two cycles was swapped (Cycle 0, Cycle 2, then Cycle 1). See Methods. (b) Fluorescence-Minus-One (FMO) controls were used to unambiguously determine appropriate gate placement for selected markers with continuous expression.

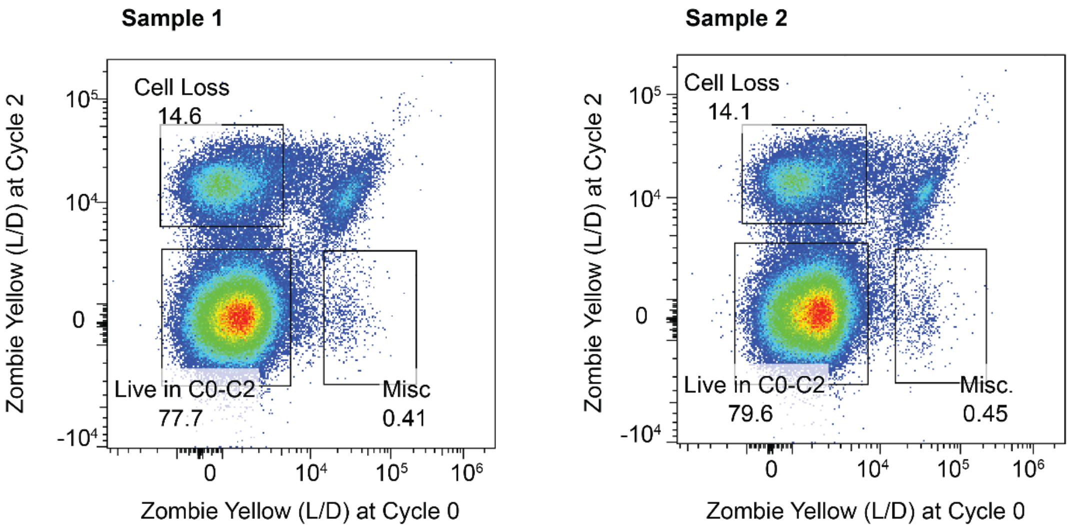

Supplementary Fig. 10 l Cell viability over the 3-cycle experiment. Cell viability by Zombie Yellow live/dead staining is measured every cycle. Plotting the viability at Cycle 0 vs Cycle 2 enables identification of cells lost ("Cell Loss") and cells that are live through all 3 cycles ("Live in C0-C2"), which are subsequently gated on for immunophenotyping analysis as shown in Fig 8. This plot also serves as a sanity check of barcode matching as <1% of those identified as "dead" in Cycle 0 were "live" in Cycle 2, which is not physically possible and can be attributed to noise or debris (gated as "Misc").

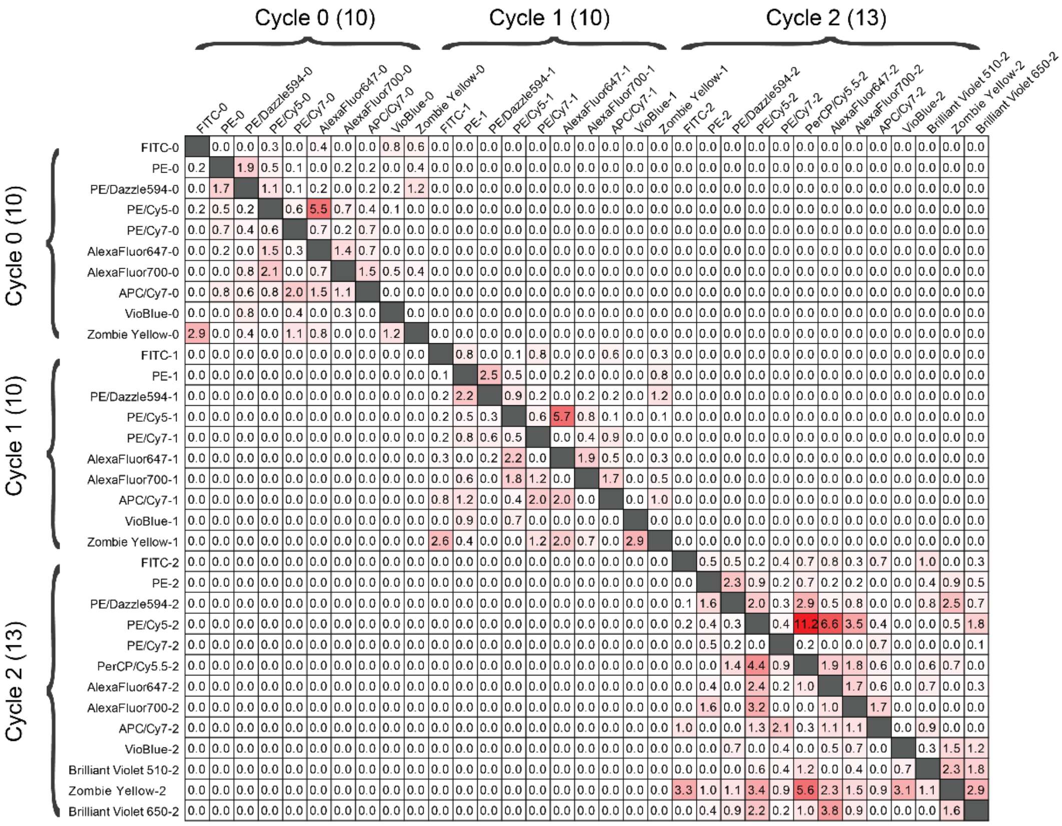

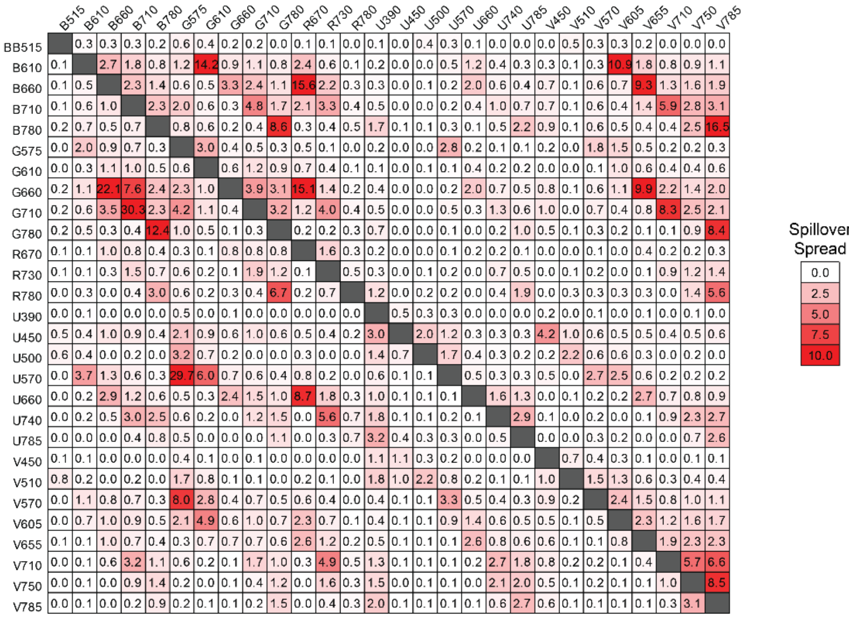

Supplementary Fig. 11 | The spillover spread matrix (SSM) shows the extent of spread between fluorophores that occurs when emitted light from each fluorophore spills into non-primary detectors. (a) SSM computed for the 3 cycle panel demonstrated in this study. No spillover or spread occurs between fluorophores used in different cycles. (b) SSM computed for a published 28-marker, conventional flow cytometry panel[40].

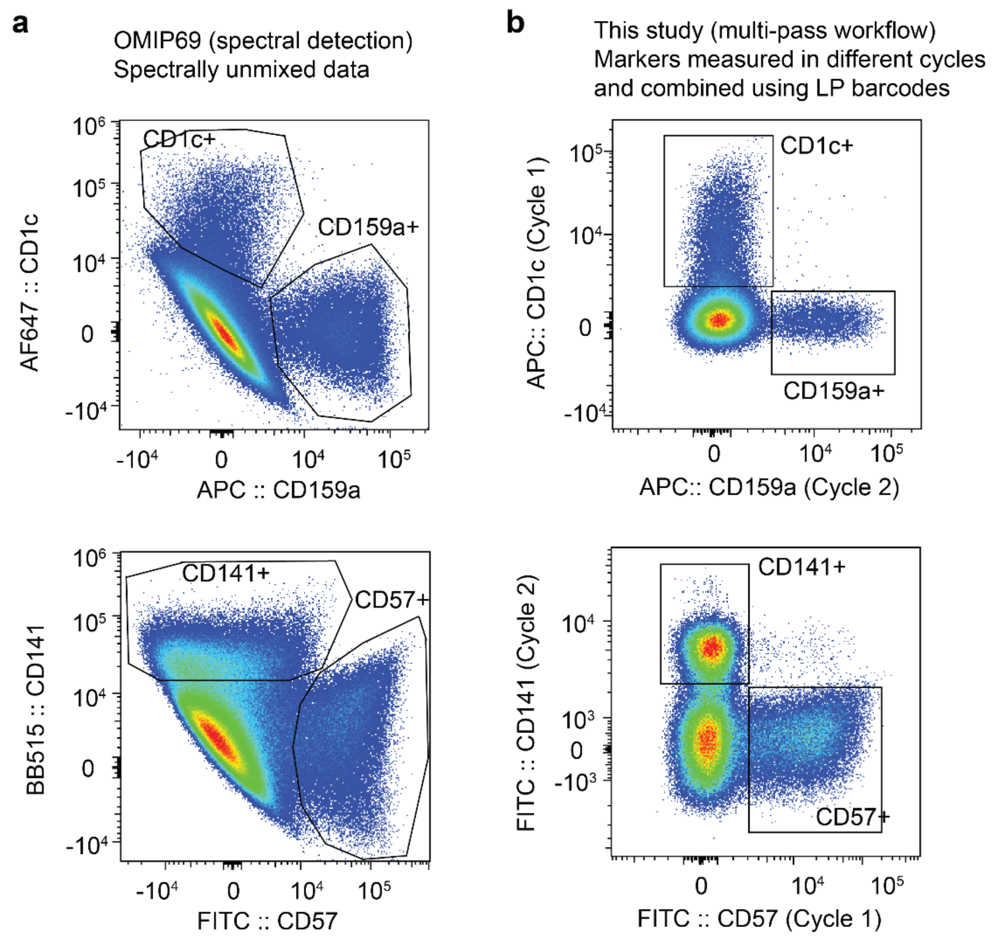

Supplementary Fig. 12 l (a) Data reproduced from OMIP-069[43], a 40-color panel acquired using full spectrum ('spectral") cytometry. Markers from two pairs of highly overlapping fluorophores are shown (AF647/APC, BB515/FITC). There is significant spread in the data (indicated by broad populations, diagonally shaped clusters) which makes it difficult to gate and identify cell subpopulations accurately. (b) Data from the study shown in this manuscript in which a 32-marker panel is split into multiple measurements with fewer colors per measurement. The plots show the same fluorophore measured in different cycles (AF647/AF647, FITC/FITC), but there is no spillover because the measurements are independent. No spreading error is observed.

Supplementary Note 1 I The total spillover spread in multi-color cytometry

When a new fluorophore is added to a panel, its fluorescence spectrum has overlap with existing fluorophores, and as a result, increases the total spillover spread (SS). The magnitude of SS, y, as a function of the number of fluorophores, x, can be expressed as:

$$\Delta y(x) = y(x) - y(x-1) = \sum_{i=1}^{x-1}(\Gamma_{ix} + \Gamma_{xi}) \tag{1}$$

where Γ_{ix} and Γ_{xi} are the elements in the rows and columns of the SS matrix containing the x-th fluorophore. Γ_{ij} is approximately proportional, but not identical, to the spectral overlap between i-th and j-th fluorophores, but the precise relation is unimportant in the analysis here.

A. Random choice of fluorophores

Let us assume that all fluorophores have identical optical properties (absorption and emission linewidths) other than their center emission frequencies. When such fluorophores are added one by one randomly, we may express the above equation in terms of expectation values, $\langle \quad \rangle$ as:

$$\langle \Delta y(x) \rangle \equiv \frac{dy}{dx} \approx 2(x-1)\,\langle \Gamma_{ij} \rangle \tag{2}$$

where $\langle \Gamma_{ij} \rangle$ represents the mean value of the matrix element. (The factor of 2 comes from the symmetry of the SS matrix, a sum along the row and the column containing the x-th fluorophore.) Eq. (2) gives

$$y = \langle \Gamma_{ij} \rangle (x-1)^2 \tag{3}$$

$\langle \Gamma_{ij} \rangle$ is roughly proportional to the ratio of the total spectral range of detection to the linewidth of fluorophores. The quadratic dependence is obvious since the number of coefficients in the SS matrix grows in 2 dimensions, where each matrix element is $\langle \Gamma_{ij} \rangle$ in this random-addition case.

B. Optimal choice of fluorophores

In practice, fluorophores do not have the same linewidths, and the mean values of $\langle \Gamma_{ix} \rangle$ and $\langle \Gamma_{xi} \rangle$ vary depending on fluorophores. Fluorophores with broader emission spectra tend to have higher mean values of $\langle \Gamma_{ix} \rangle$ and $\langle \Gamma_{xi} \rangle$ compared to fluorophores with narrower emission spectra. In this more realistic case, an experienced panel designer does not use random fluorophores but considers spectral overlap as one of the critical metrics. For a given number of markers, the user usually ends up choosing fluorophores that result in minimal SS.

In this minimal-SS condition, from Eq. (1) we can write

$$\frac{dy}{dx} = 2(x-1)\,\Gamma(x) \tag{4}$$

where $\Gamma(x) = (\langle \Gamma_{ix} \rangle + \langle \Gamma_{xi} \rangle)/2$ represents the mean overlap integral of the x-th fluorophore with the pre-populated $x-1$ fluorophores. More precisely, $\Gamma(x)$ corresponds to the sum of the matrix elements containing the x-th fluorophore (note that $\Gamma_{xx} = 0$). Under the minimal-SS protocol, $\Gamma(x)$ is a monotonously increasing function of x. (In the earlier random-choice case, $\Gamma(x)$ was a constant $\langle \Gamma_{ij} \rangle$.)

We have obtained the SS matrix from a set of 28 fluorophores used in a published T-cell panel (OMIP-060 and OMIP-068). From the matrix, we calculated $\frac{dy}{dx}$ and y using the minimal-SS protocol as a function of x from 1 to 28. Figure A shows the simulation result. The plot of y appears in Fig. 6I in the main paper.

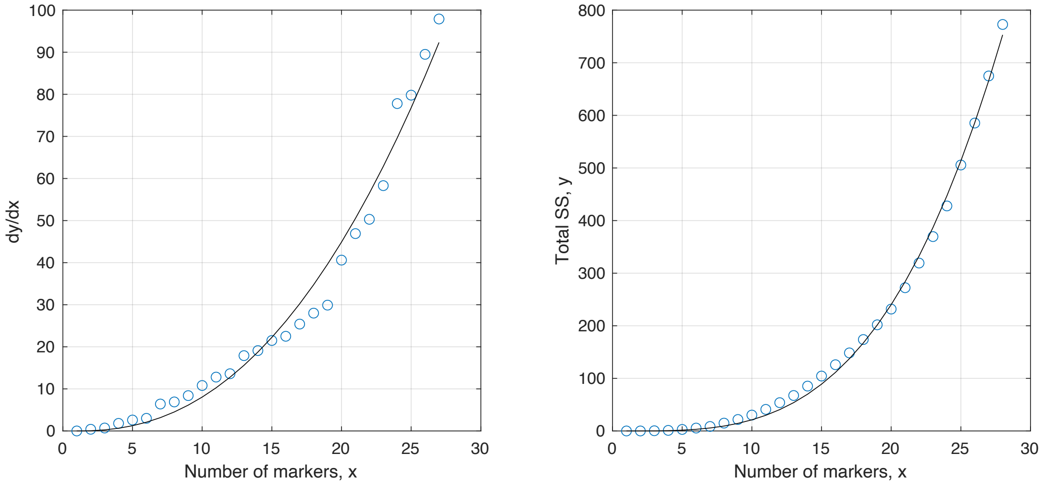

Figure A: Simulation result of the OMIP-068 panel.

The plot of $\frac{dy}{dx}$ is fit well with a single power-law function $A(x-1)^{2.3}$, which yields

$$f_\Gamma(x) \approx \frac{A}{2}\,(x-1)^{1.3} \tag{5}$$

This shows that the increase of SS by adding x-th fluorophore increases with x. This is likely due to use of non-ideal fluorophores in high-marker panels. In practice, this is driven by limited antibody availability, need to use tandem fluorophores with broader emission and absorption linewidths and instrument constraints. The increment of SS by a fluorophore may be referred to as the spillover cost of the fluorophore (analogous to the chemical potential of a molecule to the free energy of the system). For example, the spillover cost is 0, 10 A, 24.6 A, and 41.6 A for the 1st, 11th, 21st, and 31st fluorophores, respectively.
The solution of Eq. (4) is

$$y \approx B(x-1)^{3.3} \tag{6}$$

where $B = \frac{A}{3.3}$. The curve fit to the simulation data is excellent.

We find that the power-law dependence, $y \approx B(x-1)^k$, describes the characteristics of high-marker panels quite well. Figure B below shows the analysis of several other published panels, OMIP-060, OMIP-064, OMIP-067, OMIP-069, and OMIP-084. All of the results are fit reasonably well with a single power law function with an exponent in range of 2.8 to 3.5. A detailed interpretation of this finding is beyond the scope of this document.

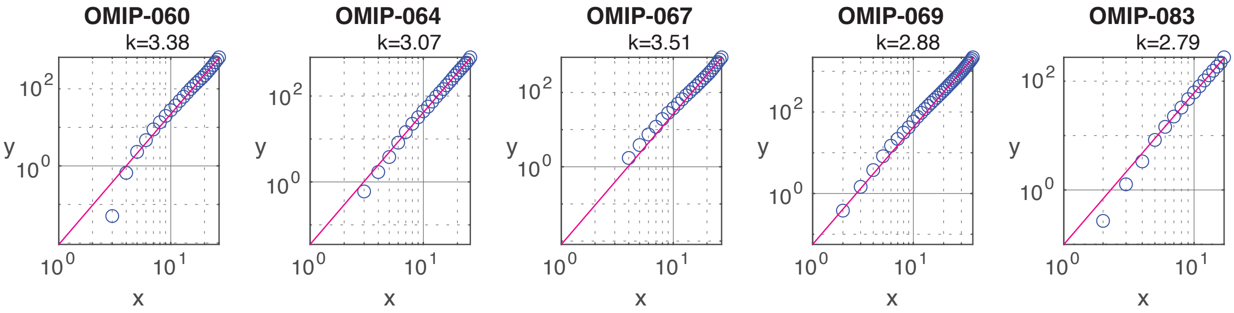

Figure B: Simulation results of various OMIP panels in the log scale. Lines, curve fit with $y = B(x-1)^k$; the best-fit k values are indicated.

For comparison, we also calculated y for the LASE's 3-cycle panels. The results are fitted with indices between 3 and 4, as shown below.

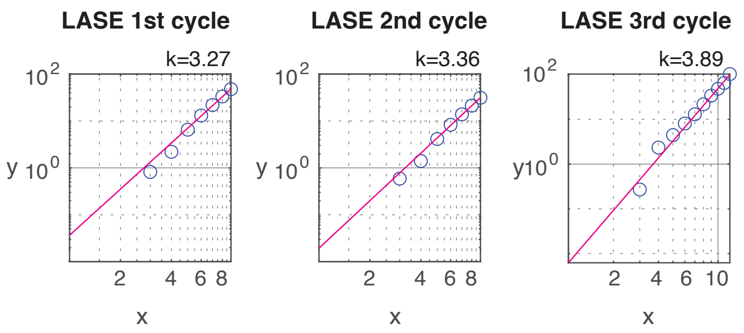

Figure C: Simulation results of the LASE 3-cycle panels in the log scale. The best-fit k values are indicated.

C. Cyclic measurement

Now, consider n-time cyclic cytometry using only x/n fluorophores. The total SSM at each cycle is $B\left(\frac{x}{n}-1\right)^{3.3}$. Since there are n cycles, the total SS is given by

$$y_n = B\left(\frac{x}{n}-1\right)^{k} \times n \quad (7)$$

For $\frac{x}{n} \gg 1,\ \ y_n \approx B\frac{(x-1)^k}{n^{(k-1)}}$. We find the ratio of the SS between the n-cyclic and non-cyclic cases to be

$$\frac{y_n}{y} \approx n^{-(k-1)} \quad (8)$$

Let us consider the above 28-marker case with Where we use k = 3.3. For $n = 3$ cycles, $\frac{y_n}{y} \approx 0.08$. This means that the SS in 3-cycle cytometry is 12.5 times lower than the SS in non-cyclic case, and this ratio is constant independent of the total number of markers.

Using $y(x) = y_n(x')$, we get $(x-1)^k = (x'-1)^k/n^{(k-1)}$ and find

$$x' = n^{(k-1)/k}\, x \quad (9)$$

For k = 3.3 and $n = 3$, we get $x' = 2.15\, x$. This means that using 3 cycles one can measure 2.15 times more markers with the same SS.

This simulation data of Eq. (7) obtained from the 28-marker panel appears in Fig. 6(e) in the main paper.

The number of fluorophores that can be used would be limited by the limited availability of fluorophores and undistinguishable spectral overlap with an existing fluorophore. Currently, the record experiment used $x = 40$ (OMIP-69, see Fig. B above). 3-cycle cytometry can extend this limit to 86. In principle, more cycles can further push the limit. With $n = 5$, up to 122 markers should be possible, requiring 24-25 fluorophores, with the same SS as the non-cyclic, 40-marker cytometry using 40 fluorophores.
