# Supplementary material for: High-dimensional multi-pass flow cytometry via spectrally encoded cellular barcoding
Source: Nat Biomed Eng. 2023 Nov 30;8(3):310–24. doi: 10.1038/s41551-023-01144-9 (PMC10963263; doi:10.1038/s41551-023-01144-9)
Supplement: Supplementary file 2 [file 41551_2023_1144_MOESM2_ESM.pdf]

nature portfolio

Corresponding author(s): Seok-Hyun Yun

Last updated by author(s): Oct 13, 2023

Reporting Summary

Nature Portfolio wishes to improve the reproducibility of the work that we publish. This form provides structure for consistency and transparency in reporting. For further information on Nature Portfolio policies, see our Editorial Policies and the Editorial Policy Checklist.

Statistics

For all statistical analyses, confirm that the following items are present in the figure legend, table legend, main text, or Methods section.

n/a	Confirmed	
☐	☒	The exact sample size (n) for each experimental group/condition, given as a discrete number and unit of measurement
☐	☒	A statement on whether measurements were taken from distinct samples or whether the same sample was measured repeatedly
☒	☐	The statistical test(s) used AND whether they are one- or two-sided<br>*Only common tests should be described solely by name; describe more complex techniques in the Methods section.*
☒	☐	A description of all covariates tested
☒	☐	A description of any assumptions or corrections, such as tests of normality and adjustment for multiple comparisons
☐	☒	A full description of the statistical parameters including central tendency (e.g. means) or other basic estimates (e.g. regression coefficient) AND variation (e.g. standard deviation) or associated estimates of uncertainty (e.g. confidence intervals)
☒	☐	For null hypothesis testing, the test statistic (e.g. F, t, r) with confidence intervals, effect sizes, degrees of freedom and P value noted<br>*Give P values as exact values whenever suitable.*
☒	☐	For Bayesian analysis, information on the choice of priors and Markov chain Monte Carlo settings
☒	☐	For hierarchical and complex designs, identification of the appropriate level for tests and full reporting of outcomes
☒	☐	Estimates of effect sizes (e.g. Cohen's d, Pearson's r), indicating how they were calculated

Our web collection on statistics for biologists contains articles on many of the points above.

Software and code

Policy information about availability of computer code

Data collection	Confocal image of LP-tagged human PBMCs was taken with Olympus' FluoVIEW software. Scanning electron micrographs were taken using ThermoFisher's Phenom ProSuite Software v2.9.0. Flow-cytometry and spectral laser-particle data were acquired using custom Python-powered software.
Data analysis	Data were analysed using custom Python scripts to extract lasing information from laser-particle emission spectra (such as peak wavelength), and to match together laser-particle barcodes. FlowJo v10.9 was used to analyse single-cell flow-cytometry data. R (ggplot2 package) was used for statistical analysis.

For manuscripts utilizing custom algorithms or software that are central to the research but not yet described in published literature, software must be made available to editors and reviewers. We strongly encourage code deposition in a community repository (e.g. GitHub). See the Nature Portfolio guidelines for submitting code & software for further information.

Data

Policy information about availability of data

All manuscripts must include a data availability statement. This statement should provide the following information, where applicable:

- Accession codes, unique identifiers, or web links for publicly available datasets
- A description of any restrictions on data availability
- For clinical datasets or third party data, please ensure that the statement adheres to our policy

The main data supporting the results in this study are available within the paper and its Supplementary Information. Source data for the figures are provided with this paper. Source data for the flow-cytometry plots are available in FlowRepository (experiment ID: FR-FCM-Z7ZJ).

Field-specific reporting

Please select the one below that is the best fit for your research. If you are not sure, read the appropriate sections before making your selection.

☒ Life sciences ☐ Behavioural & social sciences ☐ Ecological, evolutionary & environmental sciences

For a reference copy of the document with all sections, see nature.com/documents/nr-reporting-summary-flat.pdf

Life sciences study design

All studies must disclose on these points even when the disclosure is negative.

Sample size	We acquired hundreds of thousands of cells per sample, but we chose to repeat our main dataset with three different healthy human PBMC donors, 2 biological replicates each, with the 3rd donor's replicates being acquired in an additional independent experiment to further show reproducibility. Any experiments performed with less replicates are clearly indicated in the figure legends.
Data exclusions	Debris, dead cells, cell aggregates and doublets, cells showing double-positivity for multiple lineages, and (in matched samples only) cells with low-confidence matching were excluded from analysis. For the 32-marker UMAPs, 50,000 cells were downsampled from each of the six replicates from donor 3, and concatenated. Cell populations below 100 cell events and/or <0.5% of the parent population were excluded from Supplementary Fig. 5 and Supplementary Fig. 8.
Replication	All data was reproducible among each replicate and each independent experiment.
Randomization	Randomization was not relevant to the study, because we were performing all experiments using healthy donor PBMCs, which we considered equal.
Blinding	The investigators were not blinded to allocation during data collection and analysis because they were performing the experiments themselves and were cognizant of experimental conditions, groups and outcomes. However, the data were processed through many computational algorithms, which are no impacted by the absence of experimental blindness.

Reporting for specific materials, systems and methods

We require information from authors about some types of materials, experimental systems and methods used in many studies. Here, indicate whether each material, system or method listed is relevant to your study. If you are not sure if a list item applies to your research, read the appropriate section before selecting a response.

Materials & experimental systems

n/a	Involved in the study	
☐	☒	Antibodies
☒	☐	Eukaryotic cell lines
☒	☐	Palaeontology and archaeology
☒	☐	Animals and other organisms
☒	☐	Human research participants
☒	☐	Clinical data
☒	☐	Dual use research of concern

Methods

n/a	Involved in the study	
☒	☐	ChIP-seq
☐	☒	Flow cytometry
☒	☐	MRI-based neuroimaging

Antibodies

Antibodies used	Full list of the antibodies used are provided as supplementary information.
Validation	BioLegend: "Specificity testing of 1-3 target cell types with either single- or multi-color analysis (including positive and negative cell types). Once specificity is confirmed, each new lot must perform with similar intensity to the in-date reference lot. Brightness (MFI) is evaluated from both positive and negative populations. Each lot product is validated by QC testing with a series of titration dilutions."<br><br>Beckman Coulter: "Beckman Coulter offers the largest portfolio of CE-IVD and ASR conjugated antibodies validated against clinical standards. They develop and manufacture reagents according to current Good Manufacturing Practices (cGMP), the highest quality standards in the industry, ensuring optimal antibody panel performance."<br><br>Miltenyi: "Recombinant antibodies are derived from a defined set of genes, and the production process is highly standardized. This means they are consistent in their structure and performance, leading to reproducible results. Since the gene sequence of a recombinant antibody is known, it is easy to modify the sequence to improve the properties of the antibody."<br><br>Invitrogen/eBioscience: "To help ensure superior antibody results, we've expanded our specificity testing methodology using a two-part approach for advanced verification... 1) Target specificity verification 2) Functional application validation."

Flow Cytometry

Plots

Confirm that:

☐ The axis labels state the marker and fluorochrome used (e.g. CD4-FITC).

☐ The axis scales are clearly visible. Include numbers along axes only for bottom left plot of group (a 'group' is an analysis of identical markers).

☒ All plots are contour plots with outliers or pseudocolor plots.

☐ A numerical value for number of cells or percentage (with statistics) is provided.

Methodology

Sample preparation	Cryopreserved human PBMCs were purchased from AllCells & Zen-Bio. Cells were thawed through washing 2X using pre-warmed RPMI + 20% FBS + 1% Pen/Strep. After thawing, all cells were incubated in a DNase-I solution to minimize clumping. Cells were handled and stained in a modified FACS buffer.
Instrument	Data were collected on a modified Beckman Coulter CytoFLEX S.
Software	Flow-cytometry data were collected with custom Python-powered software and analysed on FlowJo v10.9. The custom code is available from the authors on reasonable request.
Cell population abundance	Not applicable.
Gating strategy	In general, each sample was gated through FSC/SSC* then FSC-A/FSC-H* then Live* then >= LP Matching Confidence (* = for each cycle).

☒ Tick this box to confirm that a figure exemplifying the gating strategy is provided in the Supplementary Information.
